# Molecular dating and viral load growth rates suggested that the eclipse phase lasted about a week in HIV-1 infected adults in East Africa and Thailand

**DOI:** 10.1371/journal.ppat.1008179

**Published:** 2020-02-06

**Authors:** Morgane Rolland, Sodsai Tovanabutra, Bethany Dearlove, Yifan Li, Christopher L. Owen, Eric Lewitus, Eric Sanders-Buell, Meera Bose, AnneMarie O’Sullivan, Raabya Rossenkhan, Jan Phillipus Lourens Labuschagne, Paul T. Edlefsen, Daniel B. Reeves, Gustavo Kijak, Shana Miller, Kultida Poltavee, Jenica Lee, Lydia Bonar, Elizabeth Harbolick, Bahar Ahani, Phuc Pham, Hannah Kibuuka, Lucas Maganga, Sorachai Nitayaphan, Fred K. Sawe, Leigh Anne Eller, Robert Gramzinski, Jerome H. Kim, Nelson L. Michael, Merlin L. Robb, the RV217 Study Team

**Affiliations:** 1 U.S. Military HIV Research Program, Walter Reed Army Institute of Research, Silver Spring, MD, United States of America; 2 Henry M. Jackson Foundation for the Advancement of Military Medicine, Inc., Bethesda, MD, United States of America; 3 Vaccine and Infectious Disease Division, Fred Hutchinson Cancer Research Center, Seattle, WA, United States of America; 4 Makerere University Walter Reed Project, Kampala, Uganda; 5 National Institute for Medical Research-Mbeya Medical Research Center, Mbeya, Tanzania; 6 Armed Forces Research Institute of Medical Sciences, Bangkok, Thailand; 7 Kenya Medical Research Institute/U.S. Army Medical Research Directorate-Africa/Kenya-Henry Jackson Foundation MRI, Kericho, Kenya; 8 International Vaccine Institute, Seoul, South Korea; University of North Carolina at Chapel Hill, UNITED STATES

## Abstract

Most HIV-1 infected individuals do not know their infection dates. Precise infection timing is crucial information for studies that document transmission networks or drug levels at infection. To improve infection timing, we used the prospective RV217 cohort where the window when plasma viremia becomes detectable is narrow: the last negative visit occurred a median of four days before the first detectable HIV-1 viremia with an RNA test, referred below as diagnosis. We sequenced 1,280 HIV-1 genomes from 39 participants at a median of 4, 32 and 170 days post-diagnosis. HIV-1 infections were dated by using sequence-based methods and a viral load regression method. Bayesian coalescent and viral load regression estimated that infections occurred a median of 6 days prior to diagnosis (IQR: 9–3 and 11–4 days prior, respectively). Poisson-Fitter, which analyzes the distribution of hamming distances among sequences, estimated a median of 7 days prior to diagnosis (IQR: 15–4 days) based on sequences sampled 4 days post-diagnosis, but it did not yield plausible results using sequences sampled at 32 days. Fourteen participants reported a high-risk exposure event at a median of 8 days prior to diagnosis (IQR: 12 to 6 days prior). These different methods concurred that HIV-1 infection occurred about a week before detectable viremia, corresponding to 20 days (IQR: 34–15 days) before peak viral load. Together, our methods comparison helps define a framework for future dating studies in early HIV-1 infection.

## Author summary

HIV-1 infected individuals rarely know when they became infected but knowing when an infection occurred provides critical information regarding HIV-1 pathogenesis and epidemiology. Using a unique cohort in which infection was known to have occurred in a narrow interval, we investigated methods to estimate the timing of infections. Several methods suggested that HIV-1 infection typically occurs a median of one week before the infection can be detected by HIV-1 RNA testing. Going forward, we provide a strategy that can be used to elucidate the origin of an acute/early infection.

## Introduction

Taking into account the date of HIV-1 infection is fundamental for epidemiological and clinical studies, yet most HIV-1 infected individuals do not reliably know their infection dates. Since biological markers vary with extreme rapidity in the first weeks of infection, having a more precise date of infection would aid in our understanding of the first steps of HIV-1 infection.

The eclipse phase is defined as the time between HIV-1 infection and a diagnosable infection. It has been estimated to last ten to twelve days: a range of 7 to 21 days is typically cited [1–7]. The Centers for Disease Control and Prevention (CDC) states that ‘a nucleic acid test can usually tell you if you are infected with HIV-1 10 to 33 days after an exposure’ (https://www.cdc.gov/hiv/basics/testing.html). Eclipse phase estimates have been defined by cases with suspected exposure (e.g., health care workers in the 1980-1990s) or on longitudinal follow up from blood bank donors who provided plasma samples twice-weekly and were, at some point, diagnosed as HIV-1 positive (and excluded from further donation). Precisely defining the eclipse phase has been difficult because 1) newly infected individuals often do not experience symptoms, thus remaining undiagnosed for potentially long periods and 2) cohorts typically collect samples too sparsely to provide narrow windows for the eclipse phase.

Here we leverage the unique RV217 prospective cohort to better estimate the eclipse phase [8]. The RV217 cohort enrolled 3,173 seronegative high-risk individuals in four countries (Kenya, Tanzania, Thailand and Uganda) and 155 acute HIV-1 infections were diagnosed. These participants did not initiate anti-retroviral treatment (ART) at the beginning of infection and were followed for up to five years, with approximately ten study visits in the first month after diagnosis. Most importantly, the RV217 study design restricted the plausible window of infection thanks to twice-weekly HIV-1 RNA tests. Diagnosis, or Day 0, was defined as the first sample that was reactive for HIV-1 RNA, which occurred a median of 4 days after the last negative visit, making this cohort the optimal one to test existing approaches and develop new methods for eclipse phase estimation.

Molecular clock methods use the genetic divergence between sequences to extract insights into the origin and spread of diseases, transmission pathways of epidemic outbreaks or putative transmission histories [9–13]. Within a host, the sequence diversity present in samples from chronic HIV-1 infection has been used to date HIV-1 infections [14–24]. Molecular dating methods have also been used on data from acute and early chronic infection [21–23], although assessing the precision of the estimates was hindered by relatively-large intervals between the last negative and first positive HIV-1 test. The early infection time scale is relevant for HIV-1 clinical trials given that testing intervals are usually of 1 to 6 months. Being able to estimate the date of infection with confidence intervals of less than 1–2 weeks is crucial to determine how a clinical trial intervention affected the occurrence of HIV-1 breakthrough infections. As such, we know that anti-HIV antibody responses elicited by the RV144 vaccine waned rapidly in 6 months [25, 26], and infusions of broadly neutralizing antibodies are cleared within weeks in humans [27–29]. Moreover, dating estimations must be possible with data collected only a few weeks or months after HIV-1 infection, as most trial designs include ART initiation upon HIV-1 diagnosis.

Therefore, we investigated methods using HIV-1 longitudinal *env* sequence data and accompanying viral load measurements from the first six months of infection to estimate the timing of the HIV-1 infection in 39 individuals. *Env* was chosen as it has a large number of parsimony informative sites compared to other genes [30–33]. Our results showed negative dates compared to the date of HIV-1 diagnosis (which corresponds to the first detectable HIV-1 RNA test), thereby defining the time when the infection started and uncovering the duration of the eclipse phase. Several methods suggested that the infection occurred a median of a week before HIV-1 diagnosis.

## Results

### HIV-1 genomes from the first days of HIV-1 infection

**Fig 1:**
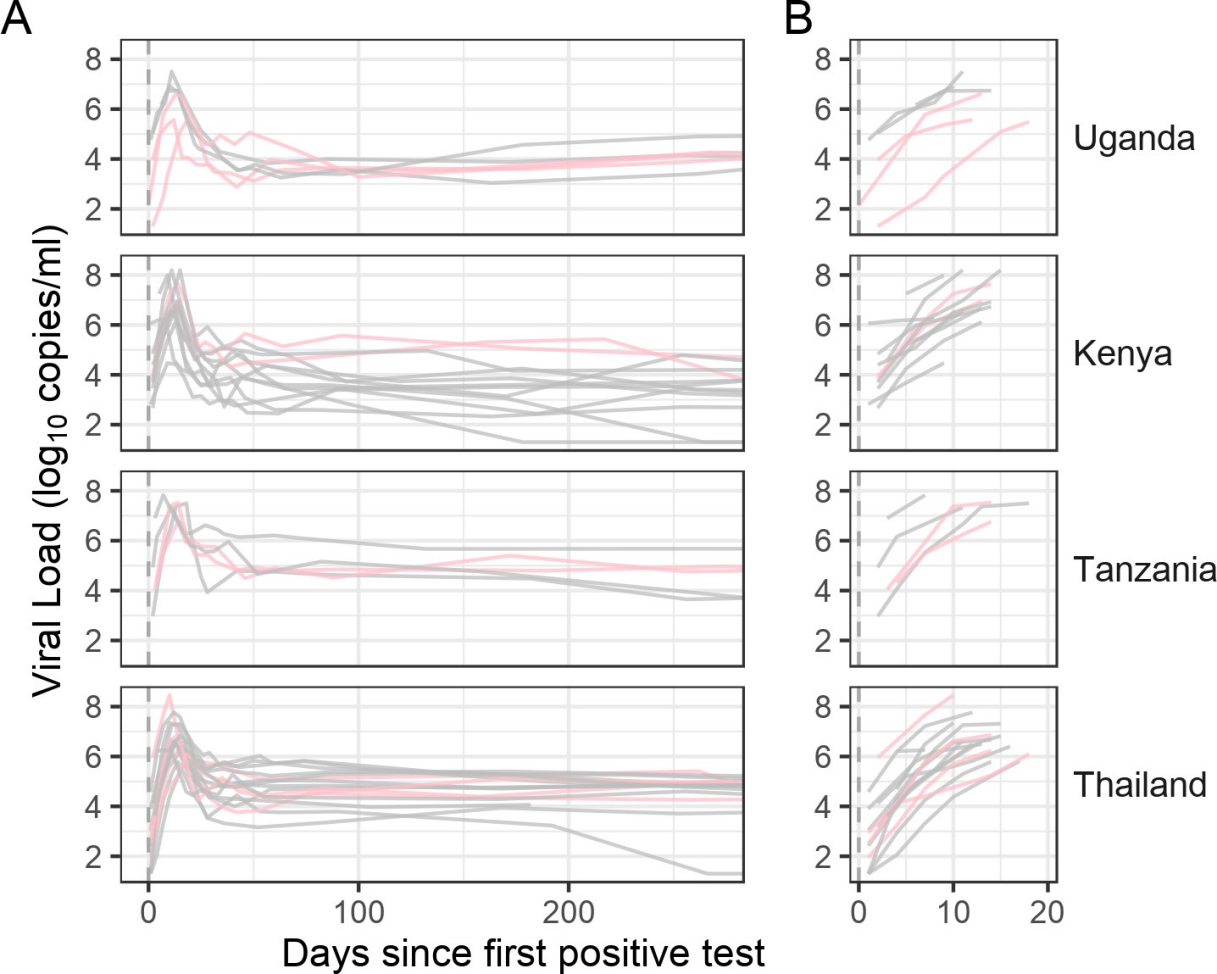
Longitudinal viral loads for 39 RV217 participants. Day 0 corresponds to the first HIV-1 positive RNA test, also referred to as diagnosis. The last negative HIV-1 RNA test occurred a median of 4 days prior to diagnosis for these participants. Panel B zooms in on the viral loads from diagnosis to peak. Participants with single founders are represented in grey, those with multiple founders in pink.

Thirty-nine HIV-1 acute infections were characterized with 1,280 HIV-1 genomes sampled from Thai participants (ten men, five transgender women and one woman) and from Kenyan, Tanzanian and Ugandan women (n = 23) in the RV217 cohort. Ten HIV-1 genomes were derived via endpoint-dilution from plasma samples collected at a median of 4, 32 and 170 days after the initial detection of viremia that occurred a median of four days after the last HIV-1 RNA negative test (**Fig 1**). At the initial sequencing time point in the first week of infection, the median viral load was 4.76 log_10_ copies/mL (range: 3.95–7.25). The highest viral load values measured in these participants, referred to as peak viral loads, occurred at a median of 13 days; the median ‘peak’ viral load was 6.74 log_10_ copies/mL (range: 4.47–8.46). The second time point sequenced, about one month after diagnosis (range: 27–42 days), occurred around the viral load nadir, which corresponded to a median of 4.55 log_10_ copies/mL (range 2.72–6.26). The last time point sequenced at six months (range: 132–261 days) showed a wide range of viral loads (median = 4.34 log_10_ copies/mL, range = 1.30–5.67) that were comparable to the set point viral load measurements (Spearman’s rho = 0.9337, p <2.2e-16; Wilcoxon signed rank test p = 0.13). Hence, large differences in viral loads were observed between participants; likewise, the genetic data showed variability across participants with a range of intra-host diversity and different tree topologies and tree lengths (**S1 Fig**). Using qualitative (e.g., tree topologies) and quantitative (e.g., intra-host maximum diversity) measures of sequence diversity as previously described [21, 34–37], we could distinguish two subgroups in our cohort: most participants (28/39) were infected with single HIV-1 founders whereas eleven participants (28%) had multiple HIV-1 founders. To detect infections with multiple founders, we also analyzed spectral density profiles derived from each participant’s maximum-likelihood phylogeny [38] [39]. We evaluated founder multiplicity based on the principal eigenvalue test, performed on the eigenvalues calculated from the weighted graph Laplacian of the distance matrix of the phylogeny for each participant (**S2 Fig**). Our test statistics illustrate the continuum of ‘multi-founderness’ of a sequence set, emphasizing the potential quandary with binary categories and, consequently, the need to investigate different methods of identification of multi-founder infections.

**Fig 2:**
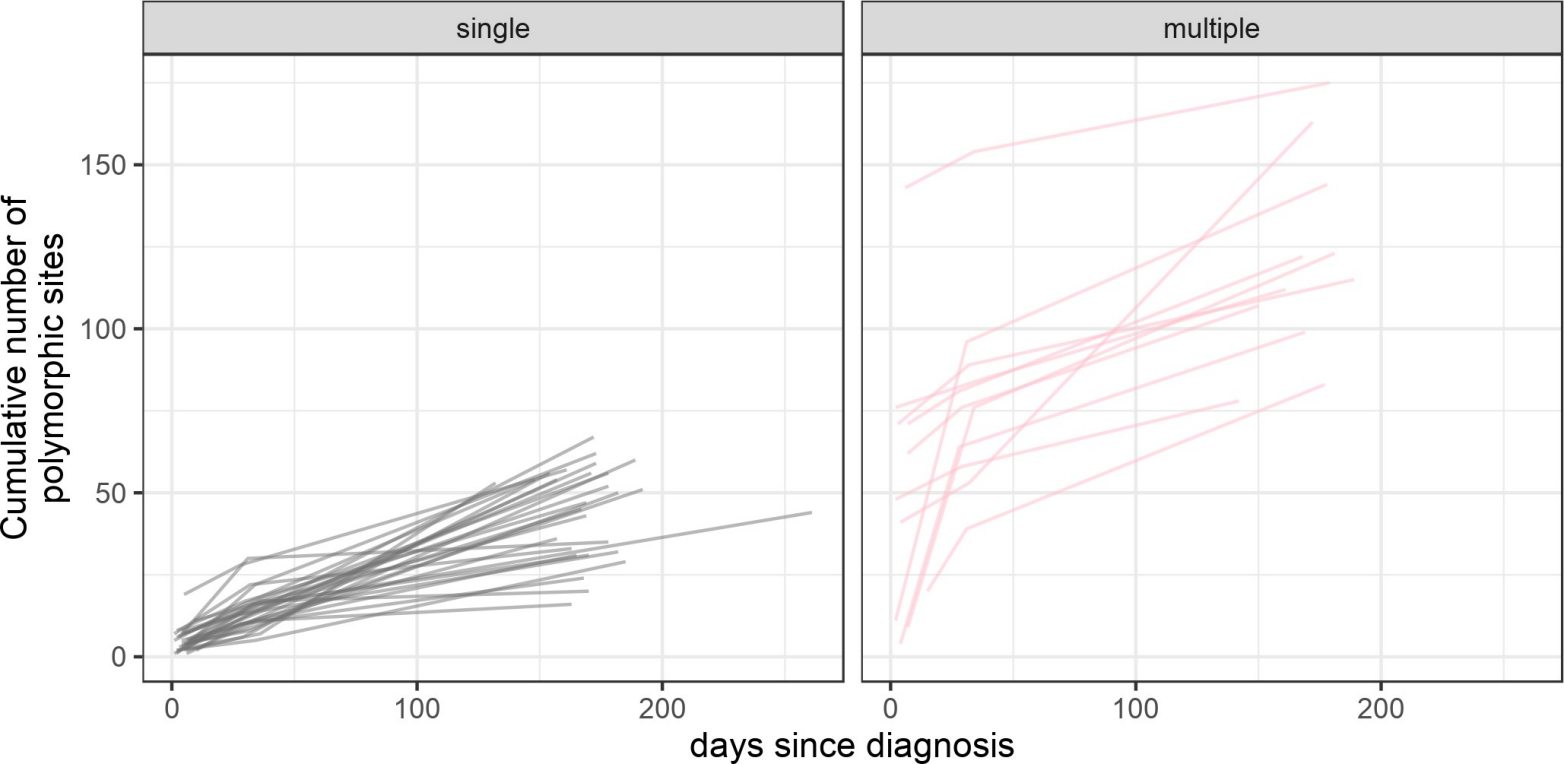
HIV-1 diversification kinetics over the first six months of infection for 39 RV217 participants. The cumulative number of polymorphic sites across all *env* sequences sampled for each participant are shown at three time points. Day 0 corresponds to the first HIV-1 positive RNA test. Participants with single founders are represented in grey, those with multiple founders in pink.

**Fig 2** shows HIV-1 diversification kinetics over the first six months of HIV-1 infection. As expected, participants with multiple HIV-1 founders showed considerably more diversity than individuals with single founders: median number of polymorphisms at baseline of 48 (range: 4–143) vs. 4.5 (range: 1–19), respectively. Considering single-founder-infections, the viral populations were homogenous at the beginning of infection with a median of 4.5 polymorphisms that increased to 9 (range: 3–24) polymorphic sites at one month and 31.5 (range: 5–56) at 6 months. Whilst the overall rate of diversification did not differ over time (**Fig 2**), it is important to note that the number of phylogenetically informative sites increased faster after the first month (**S3 Fig**). Phylogenetically informative sites are mutations shared across sequences that often correspond to sites under positive selection. This number was small in the first month (the time frame that encompassed the rise to peak viremia and the decline to the viral load nadir) but these selected mutations accumulated at a faster rate afterwards, as evidenced by the comparison of the rates of accumulation of phylogenetically informative vs. private mutations in **S3 Fig**.

Owing to HIV-1’s high mutation rates, evolutionary rates can be estimated over fairly short periods of time. Yet, it is important to ensure that there is sufficient information in the sequence set to accurately estimate substitution rates and perform Bayesian inferences [40, 41]. We excluded six participants from further molecular analyses because of insufficient phylogenetic information: four had infections with single founders (10435, 20245, 20263, 20442) and two with multiple founders (10203, 20337) (**S4 Fig**) [41]. Two participants (10435, 20263) had insufficient genetic variation, i.e., no mutations were shared across two sequences (only singleton mutations). The remaining four participants had insufficient temporal structure to their trees, i.e. no significant positive slopes between root-to-tip distance and sampling interval were found (**S4 Fig**).

### Bayesian estimates indicated a median eclipse phase of six days for infections with single HIV-1 founders

For the 33 remaining participants with sufficient phylogenetic information, we used Bayesian inference implemented in BEAST [42] to identify the best-fitting clock and population model for each participant’s *env* sequences and to estimate infection dates. Among the 24 (6 population x 4 clock) models tested, the best-fitting model differed across participants, with some model combinations never selected (**S5 Fig**). The clock and population models most frequently selected were the uncorrelated log normal relaxed clock (UCLD) (15 participants) and skyline (19 participants), respectively; the most frequent model combination was skyline with a strict clock, found in 10 participants (**S5 Fig and S1 Table**). For infections with single founders, we compared estimates obtained with either our truncated normal prior or a non-informative uniform (0,1) prior and showed that the choice of priors did not affect the age estimates. Whilst there was a difference in the estimated substitution rate for participant 20509 (which showed wide 95% HPDs under both priors), the median estimates for the age of infection differed by only 0.01 to 0.52 days for each participant (**S6 Fig**).

**Fig 3:**
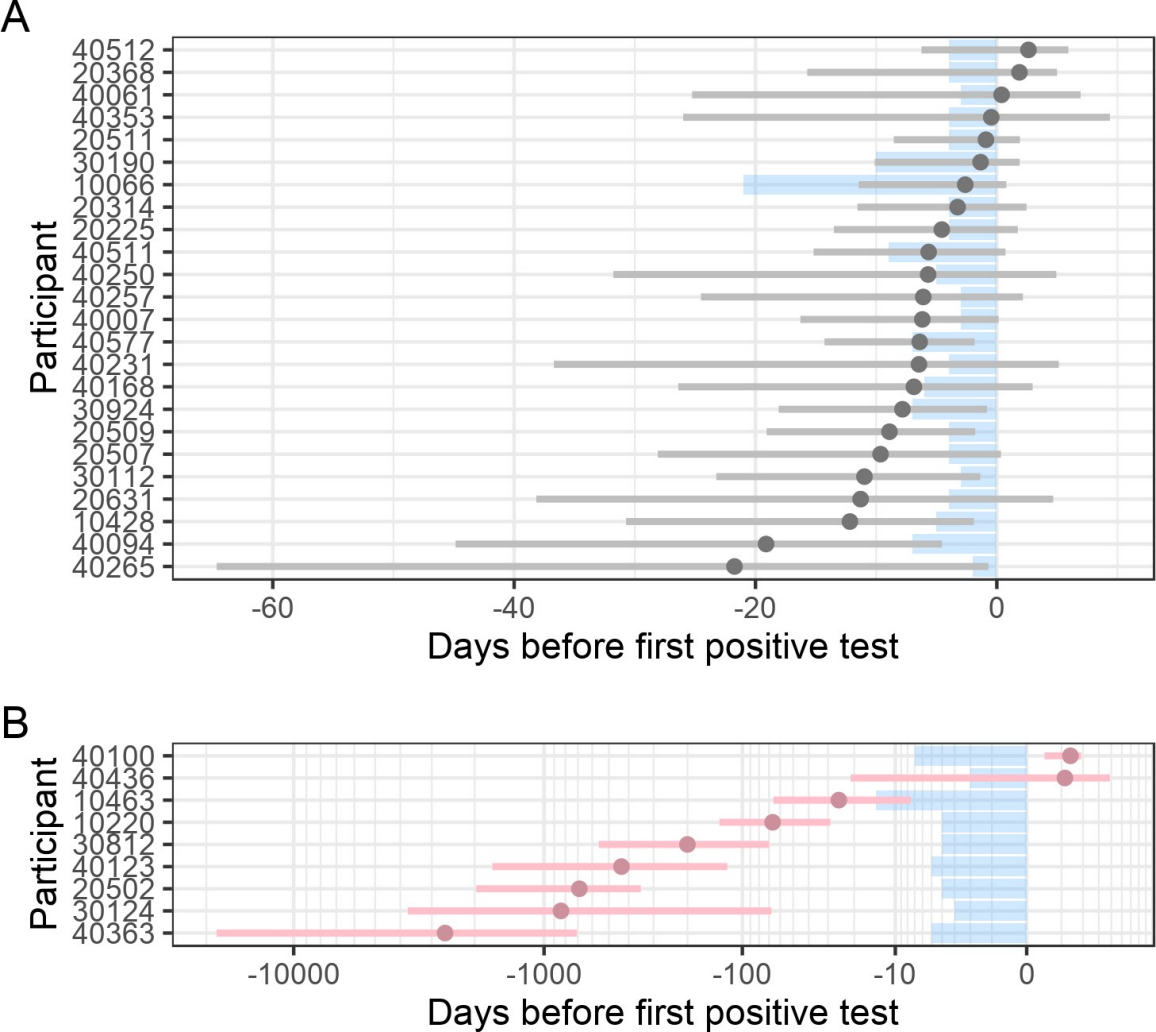
HIV-1 infection occurred a median of six days before HIV-1 diagnosis for individuals infected with single HIV-1 founder variants. The posterior medians (circle) and 95% highest posterior density interval for the best fitting model using BEAST are represented in grey for participants with single founder viruses (panel A) and in pink for those with multiple founders (panel B). The shaded blue area corresponds to the interval between the last negative and first positive HIV-1 RNA test (or diagnosis date); the time scales differ between infections with single and multiple founders. Two pregnancies were identified in acute infection: these participants had infection estimates close to their diagnosis date (1.87 and -1.35 days for 20368 and 30190, respectively).

BEAST posterior medians for the date of infection for infections with single or multiple founders ranged from 2,651 days before diagnosis to 3 days after detection of plasma viremia. Five participants had estimated infection dates after the first positive RNA test (range = 0.4–2.6 days) (**Fig 3, Table 1, S6 Fig**). Among the participants with infection estimates very close to their diagnosis date, there were two women who became pregnant around the time they got infected–the estimates were 1.87 and -1.35 days since diagnosis for 20368 and 30190, respectively. The participant with the longest estimated eclipse phase (40265) had an estimated date of infection of 22 days prior to diagnosis (95% HPD: 65–1 days). Twenty-two of the 24 participants had an estimate within 12 days before diagnosis and 13 of them within a week. We found no significant difference between participants with A1 (n = 4), CRF01_AE (n = 11) and the A1/D recombinants (n = 4) using a Kruskal-Wallis test (p = 0.56) (all other subtypes and recombinants have n = 1), or based on gender (Kruskal-Wallis rank sum test: p = 0.95) or country/continent of origin (Kruskal-Wallis rank sum test: p = 0.92, and Wilcoxon rank sum test: p = 0.84, respectively).

**Table 1:** Estimated dates of HIV-1 infection for participants with single or multiple HIV-1 founders. Median and inter-quartile range are given for the different methods investigated for all individuals (excluding those without informative temporal signal from the phylogenetic analyses) and separately for participants with single or multiple founders. The first visit from participant 30812 was excluded from Poisson-Fitter estimates (the sequence set corresponded to multiple founders and failed to go through the requirement for homogenous sequences after using the gap procedure). For hiv-founder-id, estimates were based on a subset of 28 participants, including 7 with multi-founder infections.

Method	All	Single founders	Multiple founders
Phylogenetic	n = 33	n = 24	n = 9
BEAST	-6.45 (-19.13, -2.62)	-6.14 (-9.08, -2.30)	-200.29 (-689.22, -25.42)
LSD	-100.4 (-198.81, -47.8)	-58.58 (-111.65, -43.33)	-884.49 (-1235.66, -671.28)
node.dating	-44.08 (-74.8, -32.48)	-39.27 (-61.34, -13.33)	-74.8 (-131.92, -49.18)
RTT	-37.57 (-61.13, -10.37)	-34.91 (-55.5, -10.25)	-57.47 (-145.31, -19.09)
treedater	-30.97 (-56.12, -6.67)	-32.46 (-56.5, -9.42)	-4.02 (-32.33, 2.32)
Poisson-Fitter (4 days)	-7.00 (-14.50, -4.00)	-7.50 (-14.75, -5.50)	-6.50 (-13.75, -3.50)
Poisson-Fitter (32 days)	-2.00 (-15.00, 11.00)	3.50 (-3.50, 13.00)	-36.00 (-70.00, -14.00)
hiv-founder-id	-14.00 (-16.84, -11.02)	-12.53 (-16.83, -10.84)	-14.09 (-16.49, -12.38)
**Viral load**	n = 40	n = 29	n = 11
Rmax	**-6.26** **(-10.83, -4.00)**	-7.02 (-13.07, -4.10)	-5.45 (-7.37, -3.81)
Lm	-13.15 (-20.28, -7.74)	-16.90 (-22.52, -8.74)	-11.14 (-15.96, 7.37)
Rg	-12.74 (-17.34, -9.825)	-12.84 (-17.55, -10.60)	-12.44 (-14.63, -8.67)
**Self-reports**	n = 14	n = 9	n = 4
Exposure event	-8.00 (-12.00, -5.50)	-8.50 (-13.75, -4.75)	-7.50 (-8.25, -6.50)

### Bayesian estimates on subpopulations for infections with multiple founders

Estimates for some infections with multiple founders suggested dates that were implausible (ranging from 2,651 to 85 days prior to diagnosis) as they contradicted the clinical records of these participants which showed repeat, negative HIV-1 tests (**Fig 3**). These estimates reflected the root of deep branches between variants found in their respective transmitter, i.e. the age of the infection in the transmitter. For these multi-founder infections, we split the sequences into the different founder populations and ran BEAST on the subpopulations. This improved estimates as the median date of infection was 4 days before diagnosis (IQR: 14 days pre-diagnosis to one day post-diagnosis; **S7**). However, these estimates were based on small numbers of sequences and tended to be disparate between subpopulations, e.g. more than 24 days apart in five of seven such cases.

### Poisson-Fitter indicated a median eclipse phase of eight days based on the sequences sampled four days after diagnosis

Additional molecular dating methods have been developed to maximize efficiency, yielding results in a fraction of the time required for BEAST analyses (100–1000 fold faster). We tested four such methods: Root-To-Tip (RTT), Least Square Dating (LSD), node.dating and treedater [43]. We used trees generated with IQ-TREE, comparing the best-fitting tree and the consensus from 100 bootstrap trees [41, 44–46]. Like BEAST, these methods integrate sequences sampled at multiple time points. RTT and node.dating assume a strict molecular clock while treedater allows for a relaxed molecular clock. These methods yielded estimates for the age of infection that were too large to be accurate given the participants’ clinical records: medians for single founders varied between 33 (treedater), 35 (RTT), 40 (node.dating) and 59 days (LSD), (**Table 1, S8 Fig**). To better compare these methods with BEAST, we ran node.dating and LSD while specifying a substitution rate, and treedater specifying the same prior distribution as the BEAST analyses. For the single founders, the median estimates improved slightly (treedater: 32; node.dating: 21; LSD: 22 days prior to diagnosis) (**S8 Fig**). As BEAST estimated the eclipse at 6 days, it seems that BEAST procedures were better suited than quick methods when the time scale to ascertain is in days to weeks and the sequence information content is limited.

Next, we used methods that are designed to be used with sequences from a single time point, testing alternatively the first and second time points (**Table 1**). First, we used Poisson-Fitter [34] on sequences sampled about 4–5 days after diagnosis: the estimated median date of infection occurred seven days before diagnosis, with an estimate of eight days for infections with single founders (only 1 of the 24 participants had estimates beyond three weeks) and seven days for infections with multiple founders (two participants with multiple founders failed to yield estimates). Sequences sampled one month after infection showed a median date of infection of four days after diagnosis for infections with single founders or 36 days prior to diagnosis for multiple founders. Although there is moderate agreement between Poisson-Fitter estimates from week 1 and month 1 (Kendall's coefficient of concordance = 0.71, p-value = 0.079), the estimates based on sequences from the first week seemed more accurate than those from the one-month-sequences. Finally, we tested a new method, *hiv-founder-id* [47], which scales and bounds the estimated date. Using sequences derived one month after diagnosis, the median estimated date of infection was 14 days prior to diagnosis (IQR: 17–11) (**Table 1**).

### Viral load regression indicated a median eclipse phase of six days

We used the detailed viral load measurements obtained at the beginning of infection to calculate the date of HIV-1 infection and the duration of the eclipse phase: we considered 40 RV217 participants who were enrolled when they were HIV-negative and had at least two measurements during Fiebig stages I/II (37 of the 40 participants were included in the molecular dating estimations). To ascertain the age of the infection, we estimated viral growth rates during the upslope. Since different methods can be used to calculate the viral growth rate [48], we sought to identify the most accurate method by comparing them using non-human primate (NHP) data. We tested three methods: 1) r_max_ (highest expansion rate for each subject), 2) r_g_ (linear mixed-effects model based on data from all subjects) [49] and 3) lm (individual linear model). We chose to retrogress viral loads to the time when viral loads equaled 1 copy/mL of plasma. This threshold was chosen to reflect the start of the exponential growth phase for viral loads based on previous literature [4] and comparisons with other values: the threshold of 1 copy/mL recovered the date the animals were infected more accurately than the values 0.1 and 10 copies/mL of plasma (**S9 Fig**).

**Fig 4:**
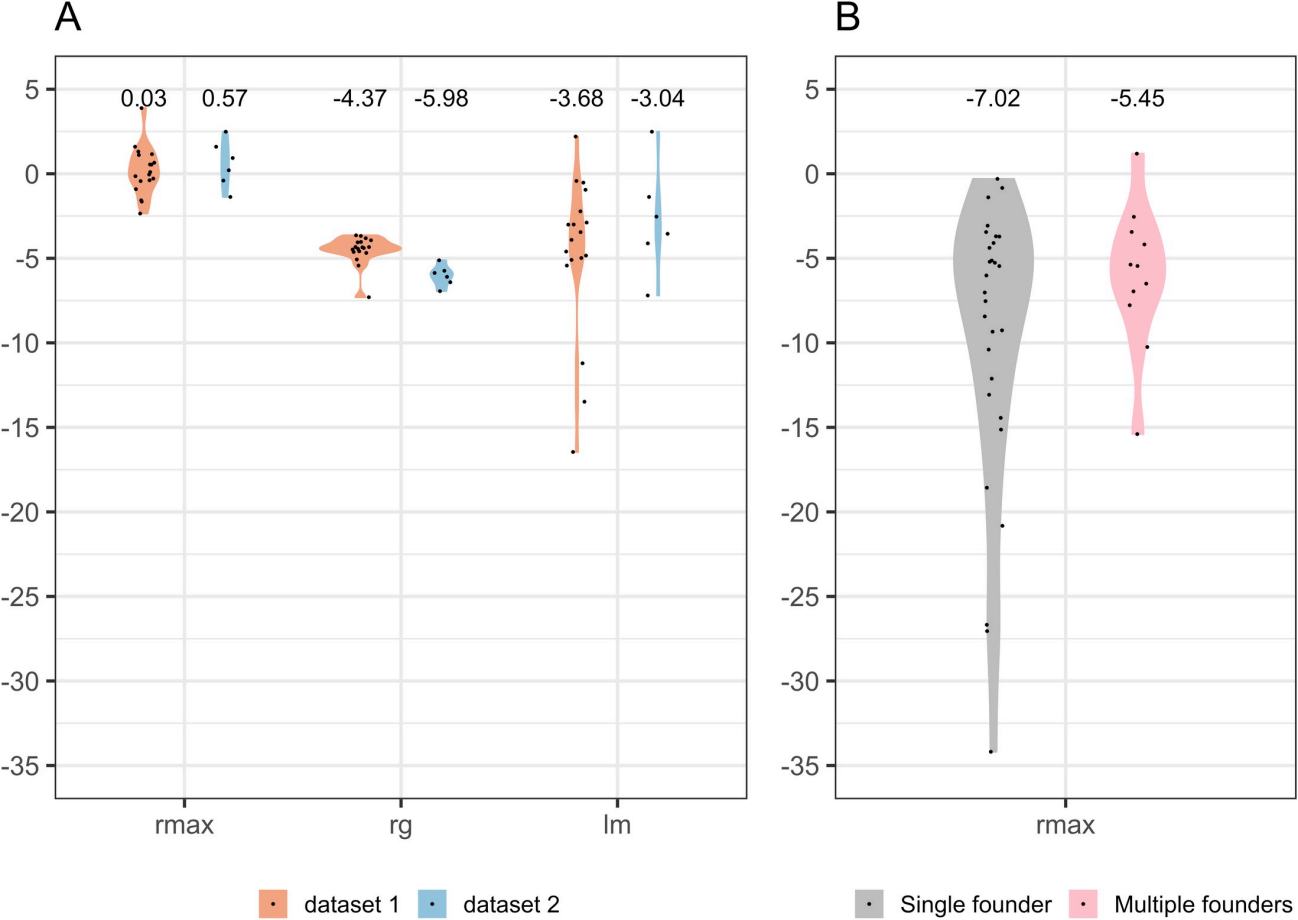
Estimated infection dates based on viral growth rates. The date when viral loads corresponded to 1 copy per mL of blood was calculated using three methods: highest expansion (r_max_), individual linear models (lm) and linear mixed-effects model based on data from all subjects (r_g_). Panel A compares the estimates in two non-human primate (NHP) cohorts with viral load data provided by Drs. Barouch (dataset 1) and Roederer (dataset 2). Panel B shows the estimates calculated with r_max_ for RV217 participants infected with single or multiple HIV-1 founder viruses. Day 0 corresponds to the day of challenge for the NHP in Panel A and to the day of the first positive HIV-1 RNA test for RV217 participants (Panel B).

To identify which of the three methods was the most accurate, we used viral load measurements from a SHIV infection study performed by Liu and colleagues [50]: this study included four groups of six animals challenged with a viral stock (SIVmac251) at four ratios of multiplicity of infection (MOI) (1:1, 1:10, 1:100 and 1:1,000). While there was no significant difference across the four MOIs, r_max_ gave the closest match to the infection date with a median of 0.03 days vs. -4.37 days using the r_g_ method or -3.68 days using the lm method (VL measurements that were below the detection limits were excluded) (**Fig 4**). We replicated this finding using data from a separate study performed by Bolton and colleagues [51]. In this case, the study included five groups of six animals: four groups received a vaccine (either an Adenovirus or a DNA/Adenovirus regimen administered either intravenously or rectally) or a placebo. Peak VL was significantly higher in the placebo group than in the vaccine groups (p-value = 0.002). Therefore, we only used animals from the placebo group to test our method. Again, r_max_ was the closest match to the day of challenge, day 0, with a median of 0.57 days while the median was -5.98 days using the r_g_ method or -3.04 days using the lm method (**Fig 4**). The rankings between methods were similar if all groups were included (**S10 Fig**).

Using the r_max_ methods for 40 participants in the RV217 cohort, the infection date was estimated at a median of 6.26 days before diagnosis (mean = 8.82, IQR = 10.83 to 4.00 days before diagnosis). Unlike the molecular dating estimates, the viral load regressions were not significantly different between participants infected with single vs. multiple founders: median = 7.02 for single founders vs. 5.45 days before diagnosis for multiple founders (p = 0.32) (**Fig 4, Table 1**). We also found that the estimates for the time of infection were shorter in Thailand than in East Africa (median = 4.61 vs. 7.40 days before diagnosis respectively, p = 0.03). Although there was a higher proportion of individuals with multiple founders in Thailand, this did not explain the geographic difference as Thai subjects with single founders also showed significantly shorter r_max_ estimates than East African participants with single founders (median = 4.61 vs. 10.40 days pre-diagnosis, p-value = 0.01). However, we found no difference between the BEAST medians between East Africa and Thailand single founders (6.19 vs. 6.14 days pre-diagnosis, respectively, p = 0.84).

### Comparison with self-reported data

**Fig 5:**
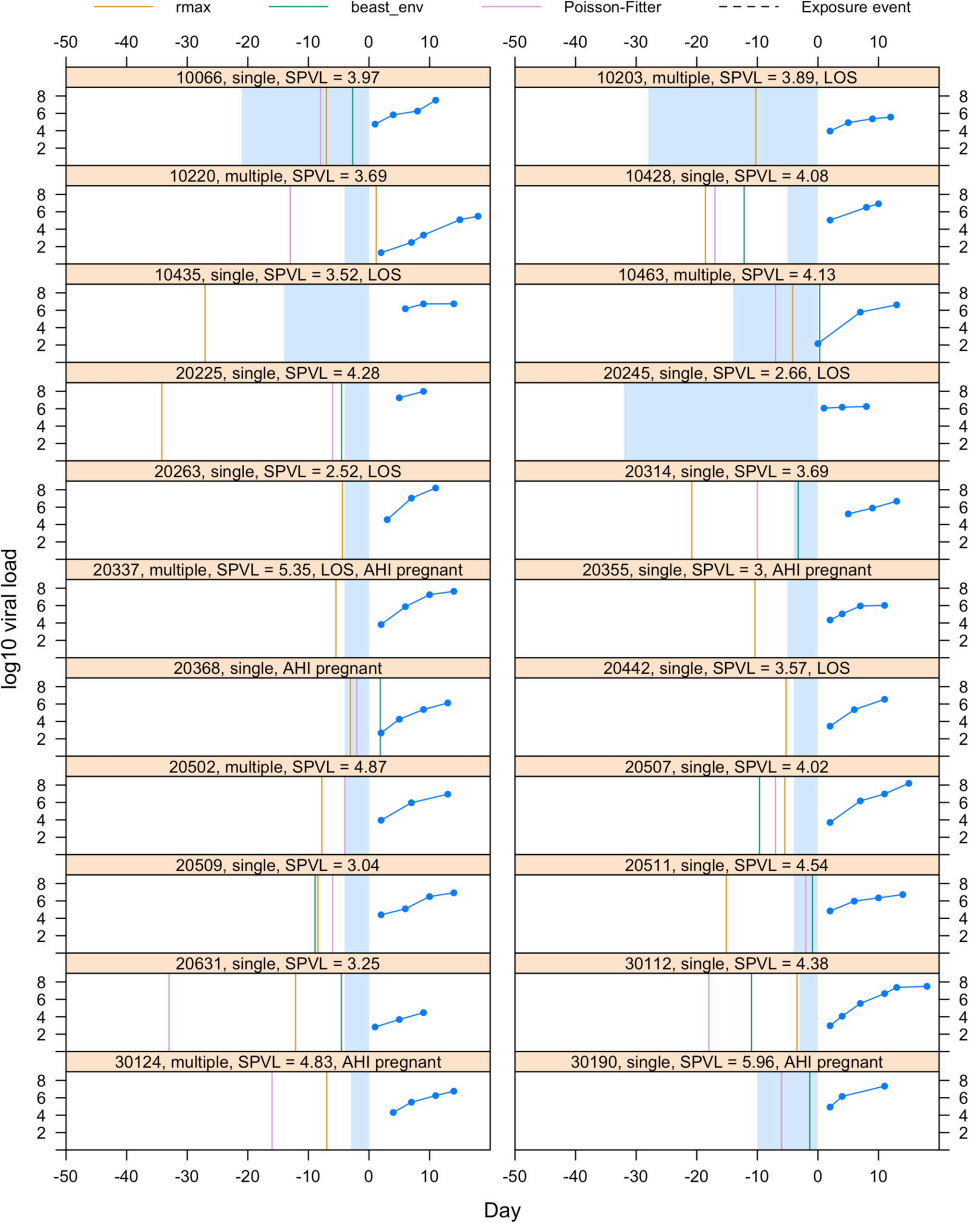
Estimates of the date of infection using three methods for 22 participants (10066 to 30190). The figures show for each individual the viral load measurements in the viral upslope (in blue), the self-reported possible transmission event (dotted black line) and the estimated dates based on molecular dating (in green), Poisson-Fitter (in plum) and viral load growth rates (in yellow). The shaded blue area corresponds to the interval between the last negative and first positive HIV-1 RNA test (or diagnosis date). The bar on top of each graph shows the participant id number along with additional information about whether they were categorized as infections with single or multiple founders, whether their sequences showed insufficient signal for phylogenetic analyses (Lack of Signal, LOS) and whether they became pregnant during this time frame, as well as their set point viral load (SPVL in log10 copies/mL).

**Fig 6:**
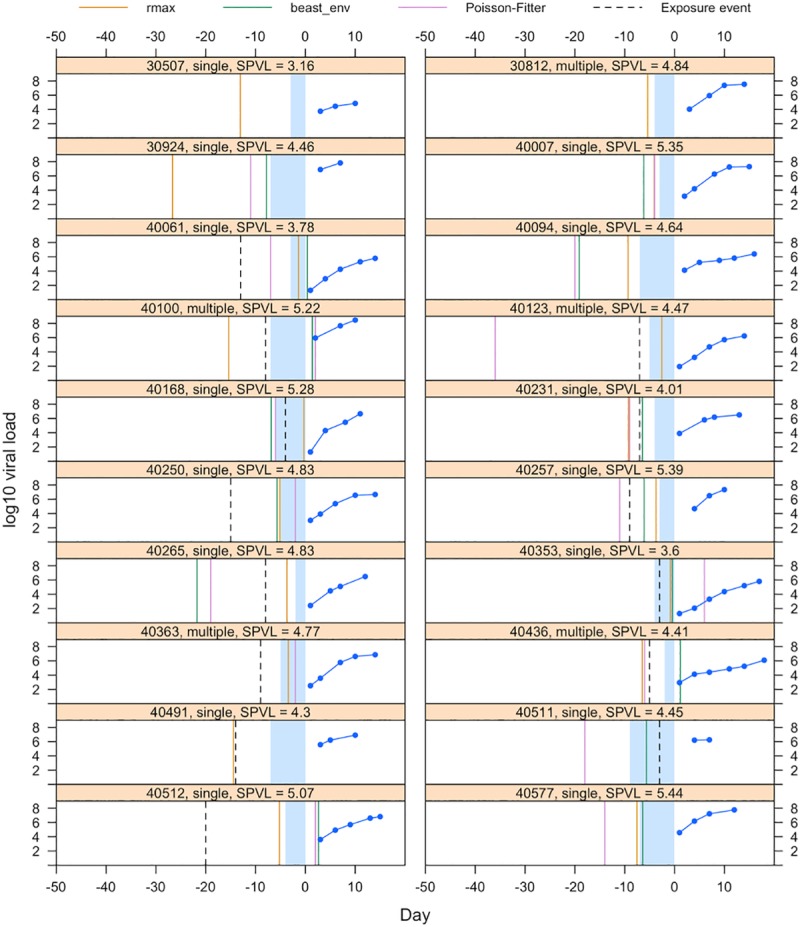
Estimates of the date of infection using three methods for 20 participants (30507 to 40577). The figures show for each individual the viral load measurements in the viral upslope (in blue), the self-reported possible transmission event (dotted black line) and the estimated dates based on molecular dating (in green), Poisson-Fitter (in plum) and viral load growth rates (in yellow). The shaded blue area corresponds to the interval between the last negative and first positive HIV-1 RNA test (or diagnosis date). The bar on top of each graph shows the participant id number along with additional information about whether they were categorized as infections with single or multiple founders, whether their sequences showed insufficient signal for phylogenetic analyses (Lack of Signal, LOS) and whether they became pregnant during this time frame, as well as their set point viral load (SPVL in log10 copies/mL).

For a subset of participants, we validated our estimates with self-reported dates of exposure. Only 14 participants could point to a single high-risk event in the few weeks preceding their diagnosis (imprecise answers, e.g. ‘in the past two weeks’, were excluded from analysis). The self-reported dates ranged from 3 to 20 days with a median of eight days before HIV-1 diagnosis (**Table 1, Figs 5 and 6**). For individuals with single founders, the self-reports were not correlated with the estimated dates of infection based on the Bayesian analyses, Poisson-Fitter or on the viral load modeling (Kendall’s test of concordance W < 0.39; p-value > 0.78). There was also no indication that the estimates based on the viral load or on the Bayesian coalescence agreed (p-value = 0.47). In contrast, Bayesian estimates were concordant with Poisson-Fitter results from the first week (Kendall’s W = 0.90, p-value = 0.008; with Poisson-Fitter estimates at one month: Kendall's W = 0.72, p-value = 0.07). **Figs 5** and 6 show the dates of infection estimated using BEAST, Poisson-Fitter (from the one week sequences) and the viral load regression; these dates are overlaid with clinical information on the participants: the window between their last negative and first positive HIV-1 RNA tests, the viral load measurements in the upslope and the self-reported high-risk exposure to HIV-1 (when available). We compared infection dates for the 23 participants infected with single founders with estimates based on three methods (BEAST, Poisson-Fitter and viral loads); although only 7 participants had estimates within five days with three methods, 21 participants had estimates within five days with at least two methods (the agreement was not restricted to molecular methods: for 14 participants, the viral load regression fell within five days of a sequence-based estimate).

## Discussion

Here we compared seven approaches to estimate the date of HIV-1 infection based on sequence data, viral loads and self-reports. Methods that yielded plausible estimates (i.e. within a month before HIV-1 diagnosis) were BEAST, Poisson-Fitter and our new VL regression (summarized in Table 1). Thanks to comparisons with a narrowly-defined window of infection, we showed that the eclipse phase of HIV-1 infection lasted about a week (six days for BEAST and the viral load modeling, seven days for Poisson-Fitter), a time frame that was slightly shorter than what is typically described in the literature (about 10–12 days). The median of six days before diagnosis corresponds to 20 days before peak viral load (IQR: 34–15 days prior); in this cohort, at peak viral load, half of the individuals had already seroconverted.

We demonstrated that samples obtained over the first half year of HIV-1 infection afforded precision on a time span of days and allowed to estimate the origin of the infection inclusive of the eclipse period (during which the virus is undetectable in the blood). HIV-1’s diversification rates were minimal at the beginning of infection but still sufficient for inferences. Our cohort matched known HIV-1 diversification patterns characteristic of the transition from demographic to selective processes after three to six weeks of infection [21, 32, 52, 53]: the accumulation of sites under selection increased after the first month, while the accumulation of singleton (random) mutations was constant over time. Although *env* diversification was still limited one month after infection, this was sufficient to impair Poisson-Fitter estimations based on these sequences. Our cohort agreed with prior studies regarding the proportion of infections with multiple founders: eleven of 39 participants (28%) were infected with multiple HIV-1 founder variants, corroborating the initial study by Keele and colleagues [21] and subsequent studies [35–37, 54–57] that showed that about a quarter of infections are established with multiple founders in men and women who have sex with men. Identifying multiple founders was important as infections with multiple HIV-1 founders led to improbably old Bayesian-estimated origins of infection that conflicted with each participant’s clinical record. Splitting the sequences into subpopulations corresponding to each founder improved the estimates but the results were not robust, suggesting that this BEAST approach would work if more sequences were available to more accurately identify and fit each founder variant.

One noticeable aspect of our dataset is the proportion of infections with insufficient diversity for Bayesian inferences. Considering how variability is a characteristic feature of HIV-1, it may be surprising that 15% of the infections we sampled had insufficient phylogenetic information for inferences; for example, there was no shared mutation in *env* after six months of infection in participant 10435. For these infections with minimal HIV-1 diversity, it is possible that larger individual sequence sets (e.g. with deep-sequencing data) would allow Bayesian estimations. As an alternative to molecular dating methods, we calculated infection dates based on viral load regression. We validated this new dating approach on two studies with challenge data from non-human primates. Irrespective of the dataset (animal or human), the best-performing method was the method based on the maximum slope during the upslope, r_max_, followed by lm and then by r_g_. Given how idiosyncratic viral load profiles were, it seems reasonable that the individual-based models would fare better than a mixed-effects model, and we suppose that, given the short duration of the viral ramp-up, the maximum slope somewhat dictated the pace of the exponential growth phase.

There are limitations to our study. First, it is conceivable that our estimates recapitulated the time frame corresponding to the systemic stage of the infection and that the initial steps of the infection between a localized infection at the mucosa and the productive dissemination were underestimated. Second, despite similar medians, tests of concordance did not show a robust correlation between the genetic or viral load-based approaches. Third, the small size of our cohort combined with several key differences between participants’ characteristics did not allow us to comprehensively evaluate the impact of certain variables on the estimates. For example, participants from East Africa were women while Thai participants were primarily men and transgender women. They were infected with various subtypes and diverged in terms of set point viral loads. Finally, only a third of the participants had described a precise high-risk HIV-1 exposure event that could be compared to the genetic or viral load regression estimates.

Our results have important implications for future clinical trials and allow us to outline a strategy for dating HIV-1 infections using data from the first weeks or months of infection. Although numerous viral load measurements are not typically available during the viral ramp-up, our work demonstrated that such data can date the origin of an infection. Hence, for future clinical trials for which information on the date of infection is crucial, investigators could choose to devise strategies to measure viral loads repeatedly at the beginning of infection. Our study indicates that twice-weekly HIV-1 tests followed by measurements every other day in the first two weeks after diagnosis could work. Further modeling work to improve our crude viral load regression method could help identify an ideal viral load sampling schedule whereas advances with HIV-1 home testing could make this approach more feasible. Given that sequence data may be more readily available, molecular dating approaches could be employed, with a strategy guided by the sampling date and the information content for each dataset. If samples were obtained in the first week(s) of infection, Poisson-Fitter could be used to date infections. For samples collected after seroconversion or beyond Fiebig stages 1–2, Bayesian coalescence methods, such as BEAST, may be better suited than Poisson-Fitter. BEAST, while computationally intensive, offered a precision that could not be matched by the faster methods we tested (RTT, LSD, node.dating, treedater). While molecular dating methods failed to give plausible results for datasets with either insufficient or complex genetic signal, increasing the depth of sequencing should improve these dating estimations. Hence, our study outlines procedures that work for dating infections based on sequences from the acute and early phases of HIV-1 infection: 1) viral load regressions if repeated viral load measurements are available in the upslope; 2) Poisson-Fitter if sequences were sampled from the first two/three weeks of infection; 3) BEAST with sufficient depth of sequencing (and multiple time points) if sequences are sampled post-peak viremia. Future work could help devise a combinatorial approach that integrates the above methods.

Importantly, the predictive power of our methodology was confirmed by the detailed information obtained from participants in our cohort, especially the narrow boundaries between the last negative visit and HIV-1 detectability. Here, the last negative visit occurred a median of four days before HIV-1 diagnosis. The known short HIV-1 infection windows were used to verify our results but was not used as priors or bounds for our estimations, warranting the use of these dating methods with confidence when similar clinical information is unavailable. As such, the methodologies we used may be critical for analyzing results from ongoing vaccine trials and from the AMP trials (HVTN 704/HPTN 085 and HVTN 703/HPTN 081). The AMP trials are testing the efficacy of the broadly neutralizing antibody VRC01 in preventing HIV-1 infections and participants receive repeat infusions of VRC01. When a new HIV-1 infection is diagnosed, estimating when it occurred with respect to the VRC01 peak/trough cycle would help determine the minimal antibody concentration that affords protection from HIV-1 infection.

## Methods

### Ethics statement

We obtained samples from the RV217 cohort [8]. The protocol was approved by the Walter Reed Army Institute of Research and local ethics review boards: the Makerere University Walter Reed Project, Kampala, Uganda; the Walter Reed Project, Kericho, Kenya; the Mbeya Medical Research Centre, Mbeya, Tanzania; and the Armed Forces Research Institute of Medical Sciences, Bangkok, Thailand. Only adult participants were enrolled. Written informed consent was obtained from all participants. We received Institutional Review Board approval to use the samples and all samples were anonymized.

### HIV-1 sequencing

Participants were selected for sequencing via single genome amplification based on several criteria: 1) the recency of the last negative visit (the median for the last negative test was 4 days before diagnosis); 2) the presence of multiple time points in the viral load upslopes; 3) the length of longitudinal antiretroviral-naïve follow up after HIV-1 infection; and 4) sample availability. Viral RNA was extracted from plasma samples using a QIAamp viral RNA kit (Qiagen); the volume of plasma ranged between 10ul for the highest VL (17,000,000 copies/mL) to 1mL for the lowest VL (3,000 copies/mL). Complementary DNA was synthesized using SuperScript III Reverse Transcriptase (Invitrogen) as instructed by the manufacturer. cDNA generated by Oligo (dT) primer was used for full genome (HXB2:789–9496) or 3’-half genome (HXB2: 4559–9496) amplification. cDNA generated by JL68RV2 (5’- CTTCTTCCTGCCATAGGAGATGCCTAAG-3’) was used for 5’-half genome amplification (HXB2: 789–5852). A nested PCR via single genome amplification [58] was employed to amplify cDNA using the Advantage GC Genomic LA kit (Clontech Laboratories, Inc., USA) as instructed by the manufacturer. Primers used for full genome amplification were MSF12B(5’-AAATCTCTAGCAGTGGCGCCCGAACAG-3’)/UNINEF (5’-GCACTCAAGGCAAGCTTTATTGAGGCTT-3’ and nested with GAG763 (5’- TGACTAGCGGAGGCTAGAAGGAGAGA-3’)/TATANEF (5’-GCAGCTGCTTATATGCAGGATCTGAGGG-3’) in the second round. Primers used for 5’ half-genome were MSF12B/JL68R V2 nested with GAG763/TAT AD’ 5’- TTCCCGGRTGKTTCCAGGGCTCTA-3’. Primers used for 3’half-genome were POLJV2 (5’- GAA GCYATGCATGGACAAGTR GA-3’)/UNINEF7' nested with POLK3 (5’- TAAARYTAGCA GGAAGATGGCCAGT-3’)/TATANEF. PCR products were purified and sequenced using an Applied Biosystems 3730 DNA Analyzer.

### Sequence curation

Sequences were annotated with sampling dates, aligned with MAFFT and edited manually in Mesquite [59, 60]. Hypermutated sequences identified via the Hypermut tool at LANL were excluded from further analysis (https://www.hiv.lanl.gov/content/sequence/HYPERMUT/hypermut.html). Sequences were partitioned according to codon position, and partitions assigned the best fit GTR sub-family model based on the Bayesian Information Criterion (BIC) implemented in PartitionFinder v2.1.1 [61]. These partitions and associated substitution models were then used as the basis for the phylogenetic analyses below. The number of phylogenetically-informative sites was calculated using the pis function in the phyloch package in R [62]. The rate of accumulation of polymorphic and phylogenetically-informative sites over time was calculated as:

$$\frac{\left(number\;of\;sites\;at\;visit\;j\right)–\left(number\;of\;sites\;at\;visit\;i\right)}{\left(number\;of\;days\;between\;visits\;i\;and\;j\right).}$$

The temporal signal in each individual alignment was evaluated using root-to-tip (RTT) on phylogenies reconstructed with IQ-TREE [45]. RTT was implemented according to Temp-Est [41] using the rtt function in the ape package in R to root the phylogeny according to the maximum correlation between tip sampling time and distance to root [62, 63]. Linear regression was used to estimate the time of the most recent ancestor and the substitution rate; a significant positive root-to-tip slope was used as evidence of sufficient temporal signal for molecular dating analyses.

### Identification of infections with multiple HIV-1 founders

Each intra-host dataset was used to identify HIV-1 infections with single or multiple founders, based on the inspection of sequence alignments and tree topologies, the results of Poisson-Fitter, and the analysis of different measures of HIV-1 intra-host diversity, including the maximum pairwise distance at each time point and the proportion of shared versus private mutations (that is, those found in only a single sequence) as previously described [21, 34–37]. Single vs. multiple founder assignments were based on 30 HIV-1 sequences derived by SGA and were thus not sensitive to rare variants. The inspection of sequence alignments was facilitated by the use of Highlighter on the HIV database at LANL (https://www.hiv.lanl.gov/content/sequence/HIGHLIGHT/highlighter_top.html) and InSites at the DiveIn website at the University of Washington (https://indra.mullins.microbiol.washington.edu/DIVEIN/insites.html) [64]. Additionally, we computed spectral density profiles for maximum-likelihood phylogenies constructed from whole genomes with IQ-TREE [46] for 38 participants. Profiles were computed by calculating the eigenvalues from the weighted graph Laplacian of the distance matrix of the phylogeny [38]. The profiles of eigenvalues for each participant were clustered based on Jensen-Shannon distances [65] using an unbiased hierarchical approach with bootstrap probabilities calculated at each node. We then evaluated the founder multiplicity of each participant based on the principal eigenvalue test [38, 39], where the threshold between single and multiple founders was determined by the median criterion (the median principal eigenvalue plus 0.5 x standard deviation squared), the jump criterion (the position of the largest discrepancy between consecutive ranked principal eigenvalues), and the partition criterion (clustering on the principal eigenvalue by partitioning around medoids [66]). Finally, we estimated the spectral density profile summary statistics for each participant: principal eigenvalue (lambda*), the skewness of the profile (psi), and the peak height of the profile (eta). We plotted participants into a three-dimensional space defined by these statistics and compared statistics between single- and multi-founders. Differences in statistics between groups were calculated using paired difference tests.

### Bayesian relaxed-clock dating

Joint estimation of the molecular dates, rates of evolution and phylogeny was performed for each participant using BEAST v1.8.2 [42]. We fitted all combinations of six different tree priors–the constant population, exponential growth, and skyline coalescent models and the constant rate birth-death model [67, 68]–with four clock models: strict clock (strict), uncorrelated lognormal relaxed clock (UCLD), uncorrelated exponential relaxed clock (UCED) and the random local clock (RLC) [69, 70]. Model selection was performed using stepping-stone sampling to assess the relative goodness-of-fit between models based on the marginal likelihood score [71]. Stepping stone sampling estimates the marginal likelihood by using a set of power posteriors to bridge from the prior to posterior. Following the recommendation by Xie and colleagues [71], and since followed by Baele and colleagues [72, 73], we selected the power values, *β*, on the path from the prior to posterior using 100 evenly spaced quantiles from the Beta(*α*,1.0) distribution with 𝛼 = 0.3. This was implemented in BEAST v1.8.2, with a chain length of 1,000,000 [72, 73]. The best fitting model was chosen as the one with the highest estimated marginal likelihood.

The substitution rate prior was set as a normal distribution with a mean of 2.24 x 10^−5^ substitutions per site per day, with a standard deviation of 0.1 and truncated with bounds of 0 and 1. The mean was derived from a within-host estimate by Lemey et al. [74] in the C2V5 region of the envelope converted from a timescale of years to days. For the constant coalescent model, a lognormal prior with log(mean) of 0 and log(standard deviation) of 1 was used for the effective population size, and for coalescent exponential and skyline models, a uniform prior between 1 and 1.0 x 10^100^ was used. All other priors were left as the default values given in BEAUti v1.8.2. Priors used for the birth-death model were a uniform prior between 0 and 100,000 for the birth rate, a uniform prior between 0 and 1 for the relative death rate, a uniform prior between 0 and 100 for the rate of sampling through time and a uniform prior between 1 and 1.0 x 10^100^ for the time the lineage originated.

BEAST was run for 250 million generations, with a thinning interval of 10,000 iterations. MCMC traces were verified for convergence using R [63] and the coda package [75]. The first 10% of each run was discarded for burn-in, with the effective sample size for each parameter verified to be above 200.

For infections with multiple founders, sequences corresponding to different founder variants were also analyzed separately. For each participant, the Gap Procedure [76] was used on the sequences from the first and second time points to cluster the sequences and identify founders. From these, a consensus was derived for each founder and used to probe to which founder each sequence from the 6-month time point belonged to (based on counts of the raw number of mutations from each consensus). Datasets that had less than 5 sequences, sequences from only a single visit, and/or no phylogenetically informative sites were excluded from further analysis (n = 11/29 datasets). For the remaining datasets, phylogenies were reconstructed using IQ-TREE and RTT was used to verify that there was significant temporal signal as described in the sequence curation section above. For all datasets with significant temporal signal, the BEAST procedure described above was repeated on the different sequence sets.

### Rapid molecular dating methods

Maximum likelihood (ML) trees for the envelopes of each participant were obtained with IQ-TREE [46] using the best-fit partitions and substitution models found through PartitionFinder [77]. Consensus trees were calculated from 100 bootstrap trees. We applied methods for calibrating the maximum likelihood trees into calendar time: root-to-tip (RTT), least squares dating (LSD), node.dating and treedater [41, 43–45]. Before applying these methods, trees were checked for polytomies, which were resolved into randomly ordered dichotomies with branch lengths set to 1.0 x 10^−10^ (zero branch lengths gave computational issues with node.dating). RTT was implemented using Temp-Est [41] as described above. The rooted tree and mutation rate obtained from RTT were used as input for node.dating. For both node.dating and RTT, the estimated mutation rate was considered regardless of whether it was significantly different from zero. For LSD, the -r option was used with the unrooted trees, which means that temporal precedence constraints were used for both the tree rooting step and the subsequent re-calibration. Treedater was run using a minimum branch length of 0.001, and 10 trees searched for the optimal rooting position.

### Poisson-fitter

Poisson-Fitter [34] analyzes the distribution of hamming distances between sequences from an homogeneous dataset and estimates the best fitting Poisson distribution through Maximum Likelihood. It also tests whether a phylogeny is star-like. We analyzed sequence sets sampled at about 4 days and one month after HIV-1 diagnosis.

### hiv-founder-id

This method employs a simple linear regression model to estimate the time of HIV infection by scaling Poisson Fitter’s estimate based on the sequences from the patient at a single timepoint; the results are constrained to the known window of infection [47]. Here we set this window to 0–60 days prior to diagnosis so that the predictive power of this approach could be tested in a similar way for all methods.

### HIV-1 testing and viral load monitoring

Samples were tested twice weekly with a qualitative HIV-1 RNA test (APTIMA). Day 0 was defined as the day on which the first blood sample was reactive for HIV-1 RNA with the APTIMA test. Plasma HIV-1 RNA levels were subsequently measured with the Abbott Molecular RealTime HIV-1 Assay (m2000 RealTime System, Abbott Molecular).

SIV/SHIV viral load data were obtained from two challenge studies in non-human primates (NHP) where the true infection day is known (challenge day, set as day 0). The first dataset (provided by Dr. Dan Barouch [50]) corresponds to a 24-animal study in which animals were challenged intra-rectally. The SIVmac251 challenge stock, which had a concentration of 109 RNA copies/ml, was inoculated at a 1:1, 1:10, 1:100 and 1:1,000 dilution with 6 animals per group. Viral load measurements were taken at days 0, 1, 2, 4, 7, 10, 14, 21, and 28 post-infection; VL measurements that were below the detection limits were excluded from the growth rate calculations. The second dataset (provided by Dr. Mario Roederer [51]) corresponds to a 30-animal study in which animals received either a vaccine (Adenovirus or DNA/Adenovirus) or a placebo with an intravenous or rectal immunization. Samples were taken at days 7, 10, 14, and 21 post-infection.

### Viral load modeling

Assuming viral load increases exponentially, we fitted linear regressions for each individual (1) and a linear mixed-effects model with random slope (2) to the log-transformed viral load data from first positive to the peak.

$$\log\;VL_{j}=\beta 0+\beta 1\;t_{j}+\varepsilon_{j}$$ (1)

$$\log\;VL_{ij}=\beta 0+(\beta 1+b_{i})\;t_{ij}+\varepsilon_{ij}$$ (2)

where *{VL ij*, *t ij}* denotes the observed viral load and sampling time for each subject I at visit j.

The viral expansion rate (r) was obtained as the slope of the regression line where r_max_ was the maximum slope between any two observations for each subject using (1), r_g_ was the subject-specific slope using linear mixed effects model (2), and lm corresponds to the slope using all observations for each subject (1).

The day of infection was estimated as the day when viral loads equaled 1 copy/mL of plasma; the first qualitative positive RNA test (Day 0) was omitted and at least two quantitative measurements in the viral upslope phase were required. We did not attempt to identify the time zero of the infection as the rate of viral growth is likely to differ before (between HIV-1 transmission and systemic dissemination) and after viral RNA reached systemic levels. Viral loads of 1 copy/mL would correspond to about 2,500 viral copies in the body (considering a volume of blood of approximately 5 liters in a typical adult). This threshold (1 copy/mL) has been used previously for other studies measuring the viral growth rate [4].

### Data visualization and availability

Plots were generated with ggplot2 and ggtree in R [78, 79]. Sequences were deposited in GenBank under accession numbers KY580473—KY580727 and MN791130—MN792579. Alignments are available at https://www.hivresearch.org/publication-supplements.

## Supporting information

S1 Table Best-fitting model for each participant. The best fitting clock and population model combination for *env* sequences corresponded to the largest estimated marginal likelihood (based on stepping-stone sampling).
(DOCX)

S1 Fig A-C Phylogenetic trees for 39 RV217 participants. Individual maximum likelihood trees were reconstructed based on *env* sequences sampled at three time points. Trees were obtained with IQ-TREE based on partitions derived from PartitionFinder and rooted using the best-fitting root from the RTT analysis. Sequences are colored to figure the time points: sequences sampled in the first week of infection are palest, and those sampled at six months are darkest. Single founders are denoted with grey tips (plots A and B), and multiple founders with pink (plot C). The participant IDs are shown on top of each tree with the first two digits corresponding to the country where participants were enrolled (Uganda: 10xxx; Kenya: 20xxx; Tanzania: 30xxx; Thailand: 40xxx). The grey horizontal bar in the bottom right corner of each plot shows the scale in substitutions per site.
(TIF)

S2 Fig The phylogenetic space of maximum-likelihood phylogenies for 38 RV217 participants. (A) Hierarchical clustering with bootstrap probabilities of Jensen-Shannon distances between spectral density profiles. Terminal branches in the dendrogram correspond to single-founders (gray) and multi-founders (pink) as described in the main text. Bootstrap probabilities > 0.95 are shown. Participant IDs are listed along the bottom of the heatmap. (B) Barplot of principal eigenvalues sorted in increasing order. Values are shifted so that the smallest value is zero. Lines indicating thresholds inferred from the median, jump, and partition criteria of the principal eigenvalue test of founder multiplicity are shown. (C) Phylogenetic space defined by spectral density profile summary statistics. (D) Boxplot of spectral density profile summary statistics between single- and multi-founders. Paired differences are significant for lambda* (p = 2.7e-4) and eta (p = 2.2e-8).
(TIF)

S3 Fig Selective HIV-1 processes after the first month of infection. The number of polymorphisms were counted across *env* sequences from each participant for two intervals: between one week and one month, between one month and six months. Private and shared mutations are shown separately. The slopes (calculated using the interval between sampling time points for each participant) were compared for infections with single HIV-1 founder variants. When considering all polymorphisms, the distribution of points on both sides of the line shows that the rate of diversification did not differ across time points. For shared mutations, which represent selected sites, the rate increased after one month.
(TIF)

S4 Fig Estimated substitution rates. The substitution rates were estimated with RTT [41] and participants are grouped based on whether there was a significant positive slope between the root-to-tip distance and sampling interval (labelled, ‘True’) or no significant slope (‘False’). Participants with single vs. multiple founders are plotted separately and on different scales as the substitution rates are higher for infections with multiple founders. The presence of phylogenetically informative sites is figured with a filled circle.
(TIF)

S5 Fig Most frequent clock and population models. The heatmap shows the proportion of individuals for which a given clock and population model was selected as the best-fitting. Counts give the number of participants for which each model was chosen. Data are presented separately for participants infected with single or multiple founder HIV-1 variants.
(TIF)

S6 Fig Substitution rate priors did not affect BEAST estimates. For infections with single founders, BEAST inferences run using either a truncated normal prior (in grey) were compared to a non-informative uniform prior (0,1) (in blue) (both under the best-fitting model). Circles show the median estimates and bars indicate the 95% HPD for the date of infection (A) and the substitution rate (B). The shaded blue area corresponds to the interval between the last negative and first positive HIV-1 RNA test.
(TIF)

S7 Fig Improved BEAST estimates on the subpopulations from infections with multiple founder variants. The posterior medians (circle) and 95% highest posterior density interval for the best fitting model are shown. The shaded blue area corresponds to the interval between the last negative and first positive HIV-1 RNA test (or diagnosis date). The number of sequences and number of polymorphic sites corresponding to each subpopulation are reported. Only subpopulations with sequences covering at least two time points, a minimum of five sequences with more than one phylogenetically-informative site, and significant temporal signal were analyzed.
(TIF)

S8 Fig Estimates of the date of infection using four sequence-based methods. Results based on treedater, RTT, node.dating and LSD are shown for both the best-fitting trees and the consensus from 100 bootstrap trees (using IQ-Tree). In Panel A, no prior information was set; for panel B, we specified the substitution rate as 2.24x10-5 for node.dating and LSD and used the truncated normal prior that we used in BEAST for treedater. Infections with single founders are shown in grey and multiple founders in pink.
(TIF)

S9 Fig Estimated infection dates based on viral growth rates using three thresholds for the initial infection. The infection dates were calculated using three methods: highest expansion (r_max_), linear mixed-effects model based on data from all subjects (r_g_), and individual linear models (lm). Viral growth rates were regressed using three initial viral load thresholds: 0.1, 1 and 10 copies/mL. Estimates are compared for two non-human primate (NHP) cohorts with viral load data provided by Drs. Barouch (dataset 1) and Roederer (dataset 2) (animals were infected at day 0).
(TIF)

S10 Fig Estimated infection dates based on viral growth rates for 30 animals immunized with a vaccine or a placebo. Animals belonged to one of five groups of six animals: four groups received a vaccine (either an Adenovirus or a DNA/Adenovirus regimen administered either intravenously or rectally) or a placebo (Bolton and colleagues [51]). Vaccine or placebo status is figured with a triangle or a circle, respectively. The date when viral loads corresponded to 1 copy per mL of blood was calculated using three methods: highest expansion (r_max_), individual linear models (lm) and linear mixed-effects model based on data from all animals (r_g_). The estimates followed the same rankings whether the animals received a vaccine or a placebo; only the placebo group was reported for the evaluation of methods (**S9 Fig**).
(TIF)
