# Supplementary material for: Molecular dating and viral load growth rates suggested that the eclipse phase lasted about a week in HIV-1 infected adults in East Africa and Thailand
Source: PLoS Pathog. 2020 Feb 6;16(2):e1008179. doi: 10.1371/journal.ppat.1008179 (PMC7004303; doi:10.1371/journal.ppat.1008179)
Supplement: S1 Table — The best fitting clock and population model combination for env sequences corresponded to the largest estimated marginal likelihood (based on stepping-stone sampling). (DOCX) [file ppat.1008179.s001.docx]

**Supplementary Table 1. Best-fitting model for each participant.** The best fitting clock and population model combination for *env* sequences corresponded to the largest estimated marginal likelihood (based on stepping-stone sampling).

| **Participant** | **Clock** | **Population** | **Marginal likelihood** |
| --- | --- | --- | --- |
| 10066 | strict | skyline | -4238.159 |
| 10220 | strict | skyline | -4323.851 |
| 10428 | strict | skyline | -4072.244 |
| 10463 | ucld | bd | -4709.288 |
| 20225 | uced | bd | -4015.707 |
| 20314 | ucld | bd | -4144.639 |
| 20368 | ucld | skyline | -3882.577 |
| 20502 | strict | skyline | -3613.629 |
| 20507 | ucld | bd | -4009.401 |
| 20509 | ucld | bd | -3771.093 |
| 20511 | ucld | skyline | -4154.091 |
| 20631 | ucld | bd | -3963.247 |
| 30112 | uced | bd | -3860.282 |
| 30124 | uced | skyline | -5323.235 |
| 30190 | ucld | skyline | -4002.558 |
| 30812 | ucld | exponential | -5061.785 |
| 30924 | ucld | bd | -4041.023 |
| 40007 | ucld | bd | -3875.826 |
| 40061 | ucld | skyline | -3966.016 |
| 40094 | strict | skyline | -4108.442 |
| 40100 | uced | skyline | -5412.745 |
| 40123 | uced | skyline | -4758.904 |
| 40168 | strict | skyline | -4083.078 |
| 40231 | uced | skyline | -4028.227 |
| 40250 | strict | skyline | -3881.943 |
| 40257 | strict | skyline | -4148.859 |
| 40265 | uced | skyline | -4013.557 |
| 40353 | strict | skyline | -3765.447 |
| 40363 | rlc | exponential | -5057.094 |
| 40436 | ucld | constant | -4675.817 |
| 40511 | ucld | bd | -3981.150 |
| 40512 | strict | skyline | -4209.128 |
| 40577 | ucld | bd | -4086.494 |
