# Supplementary figures and images for: Molecular dating and viral load growth rates suggested that the eclipse phase lasted about a week in HIV-1 infected adults in East Africa and Thailand
Source: PLoS Pathog. 2020 Feb 6;16(2):e1008179. doi: 10.1371/journal.ppat.1008179 (PMC7004303; doi:10.1371/journal.ppat.1008179)

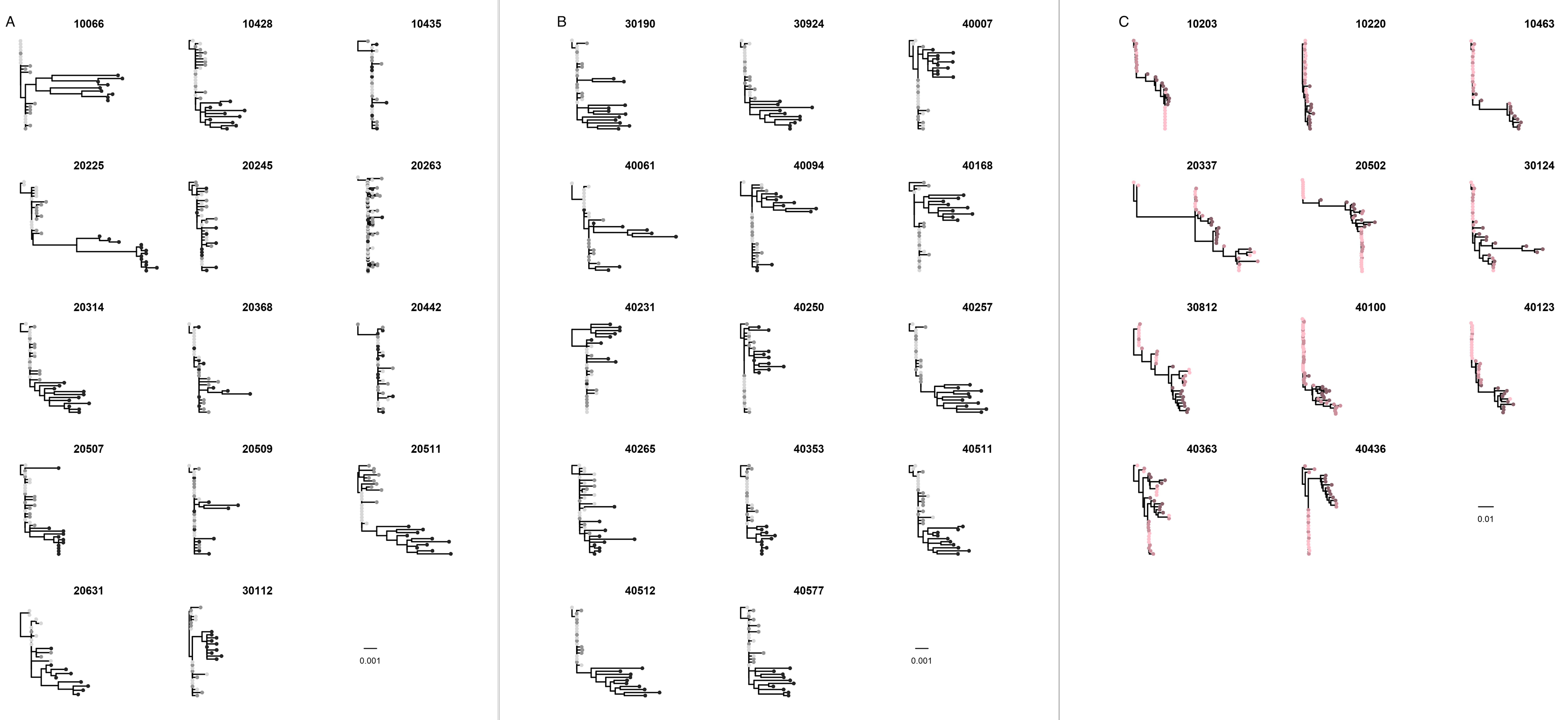

Supplement: S1 Fig — Individual maximum likelihood trees were reconstructed based on env sequences sampled at three time points. Trees were obtained with IQ-TREE based on partitions derived from PartitionFinder and rooted using the best-fitting root from the RTT analysis. Sequences are colored to figure the time points: sequences sampled in the first week of infection are palest, and those sampled at six months are darkest. Single founders are denoted with grey tips (plots A and B), and multiple founders with pink (plot C). The participant IDs are shown on top of each tree with the first two digits corresponding to the country where participants were enrolled (Uganda: 10xxx; Kenya: 20xxx; Tanzania: 30xxx; Thailand: 40xxx). The grey horizontal bar in the bottom right corner of each plot shows the scale in substitutions per site. (TIF) [file ppat.1008179.s002.tif]

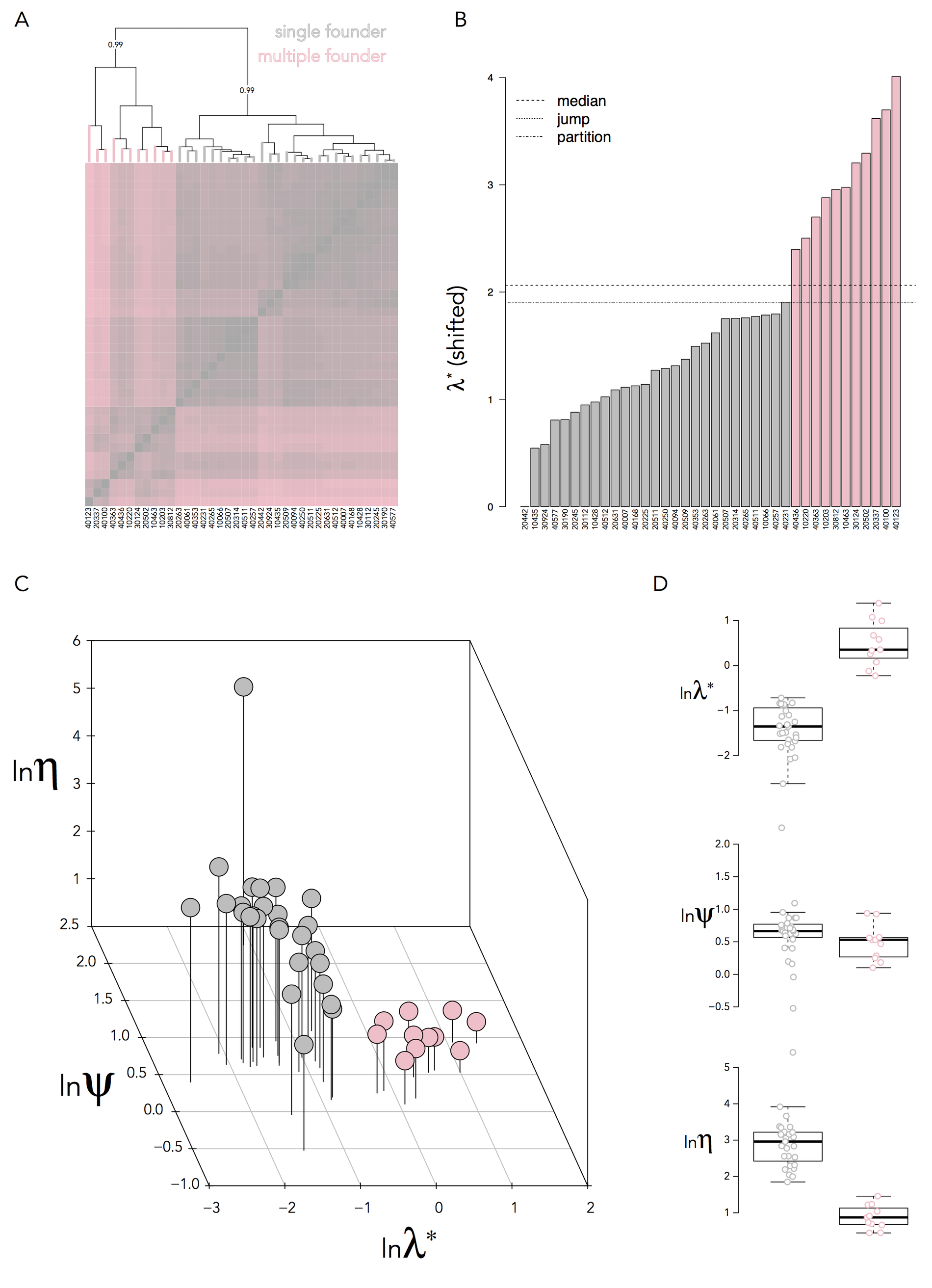

Supplement: S2 Fig — (A) Hierarchical clustering with bootstrap probabilities of Jensen-Shannon distances between spectral density profiles. Terminal branches in the dendrogram correspond to single-founders (gray) and multi-founders (pink) as described in the main text. Bootstrap probabilities > 0.95 are shown. Participant IDs are listed along the bottom of the heatmap. (B) Barplot of principal eigenvalues sorted in increasing order. Values are shifted so that the smallest value is zero. Lines indicating thresholds inferred from the median, jump, and partition criteria of the principal eigenvalue test of founder multiplicity are shown. (C) Phylogenetic space defined by spectral density profile summary statistics. (D) Boxplot of spectral density profile summary statistics between single- and multi-founders. Paired differences are significant for lambda* (p = 2.7e-4) and eta (p = 2.2e-8). (TIF) [file ppat.1008179.s003.tif]

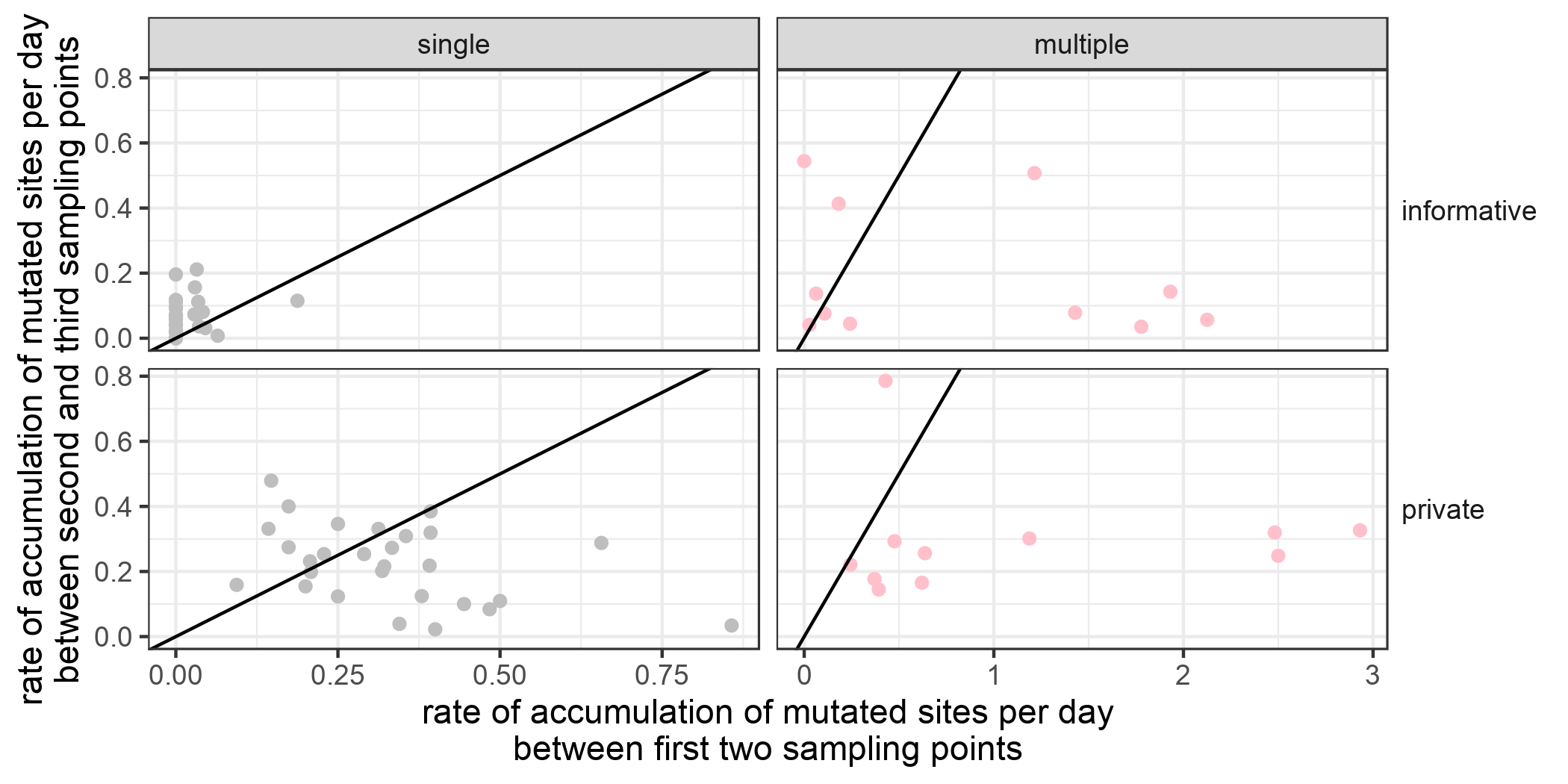

Supplement: S3 Fig — The number of polymorphisms were counted across env sequences from each participant for two intervals: between one week and one month, between one month and six months. Private and shared mutations are shown separately. The slopes (calculated using the interval between sampling time points for each participant) were compared for infections with single HIV-1 founder variants. When considering all polymorphisms, the distribution of points on both sides of the line shows that the rate of diversification did not differ across time points. For shared mutations, which represent selected sites, the rate increased after one month. (TIF) [file ppat.1008179.s004.tif]

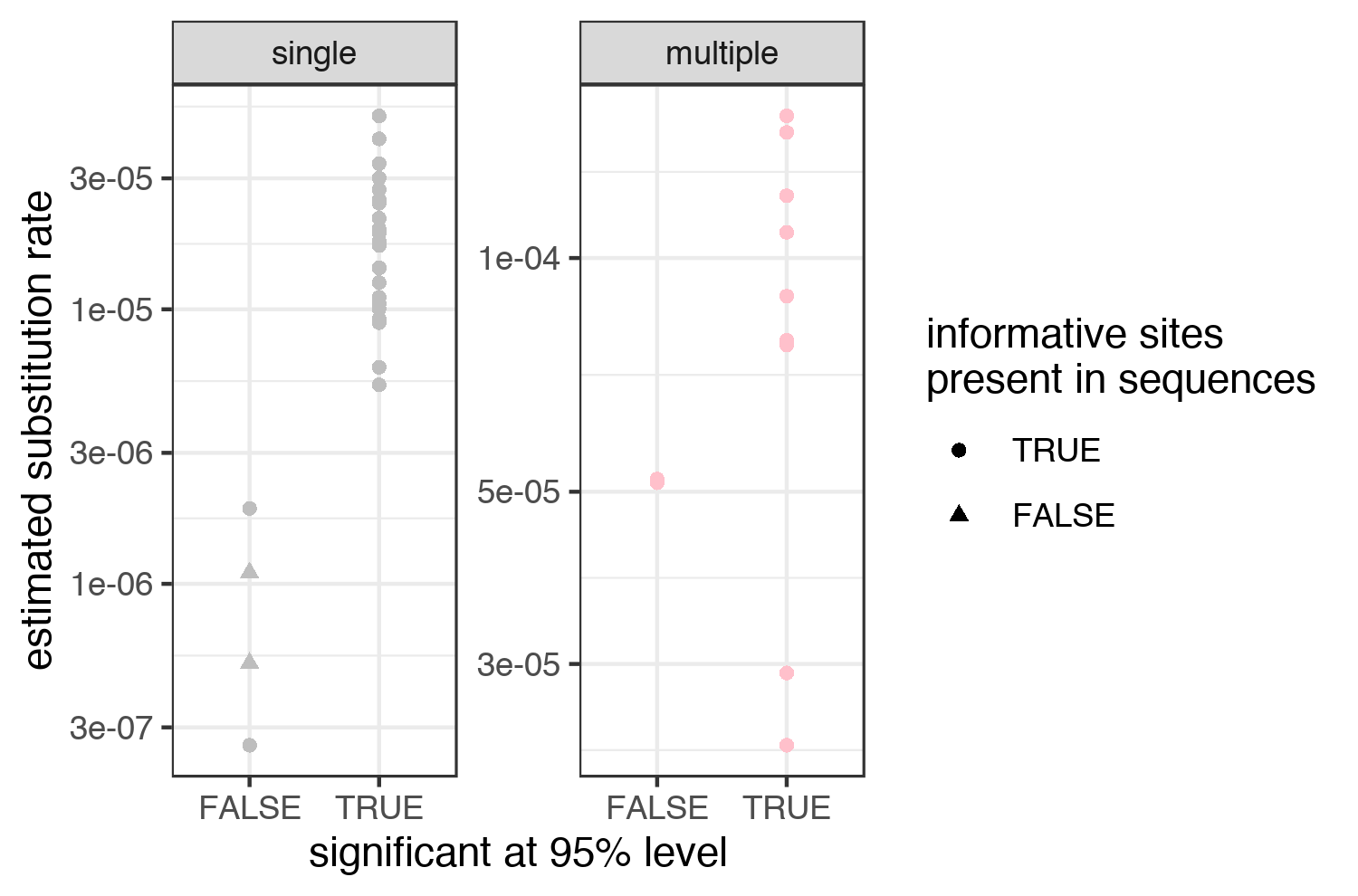

Supplement: S4 Fig — The substitution rates were estimated with RTT [41] and participants are grouped based on whether there was a significant positive slope between the root-to-tip distance and sampling interval (labelled, ‘True’) or no significant slope (‘False’). Participants with single vs. multiple founders are plotted separately and on different scales as the substitution rates are higher for infections with multiple founders. The presence of phylogenetically informative sites is figured with a filled circle. (TIF) [file ppat.1008179.s005.tif]

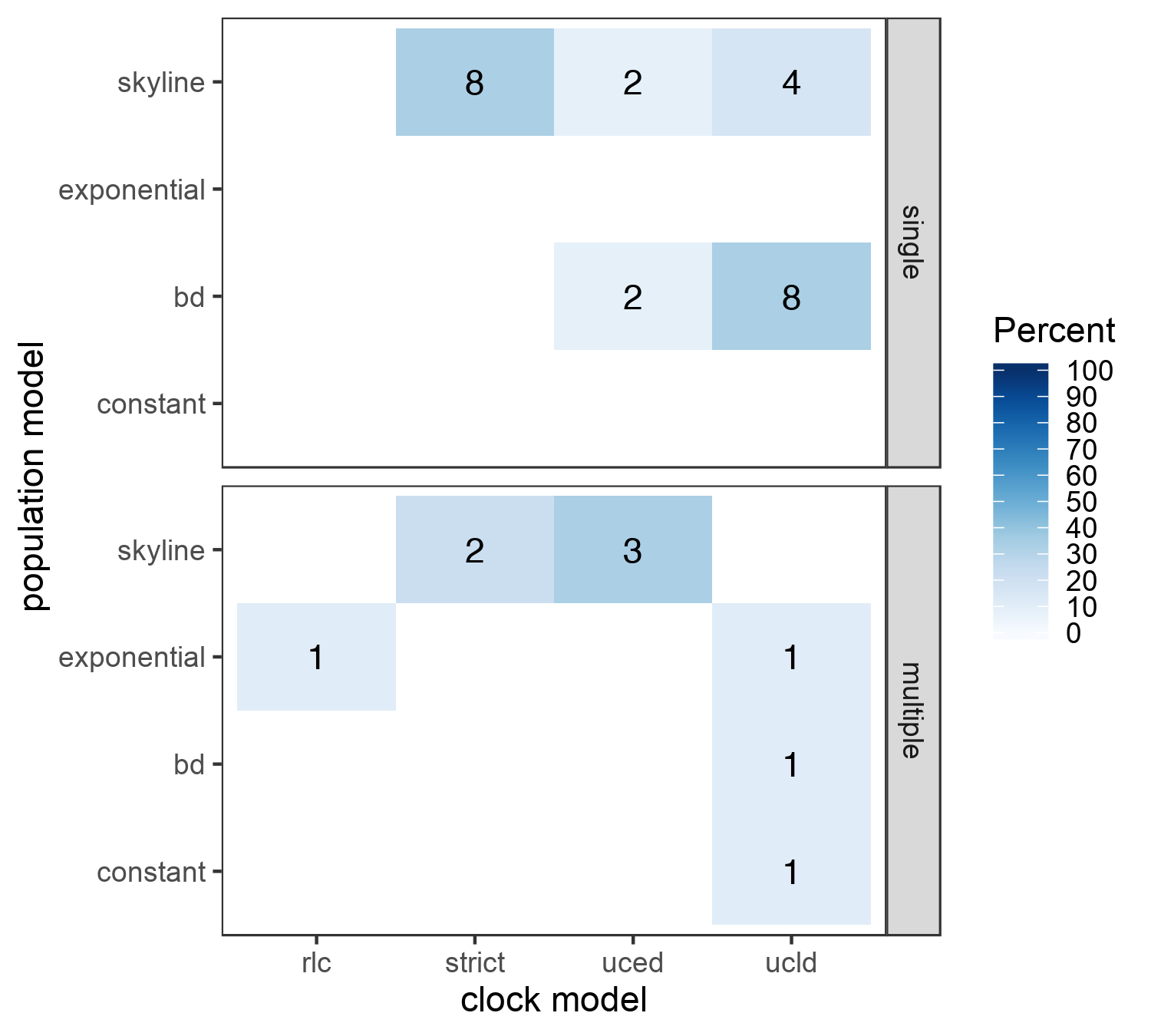

Supplement: S5 Fig — The heatmap shows the proportion of individuals for which a given clock and population model was selected as the best-fitting. Counts give the number of participants for which each model was chosen. Data are presented separately for participants infected with single or multiple founder HIV-1 variants. (TIF) [file ppat.1008179.s006.tif]

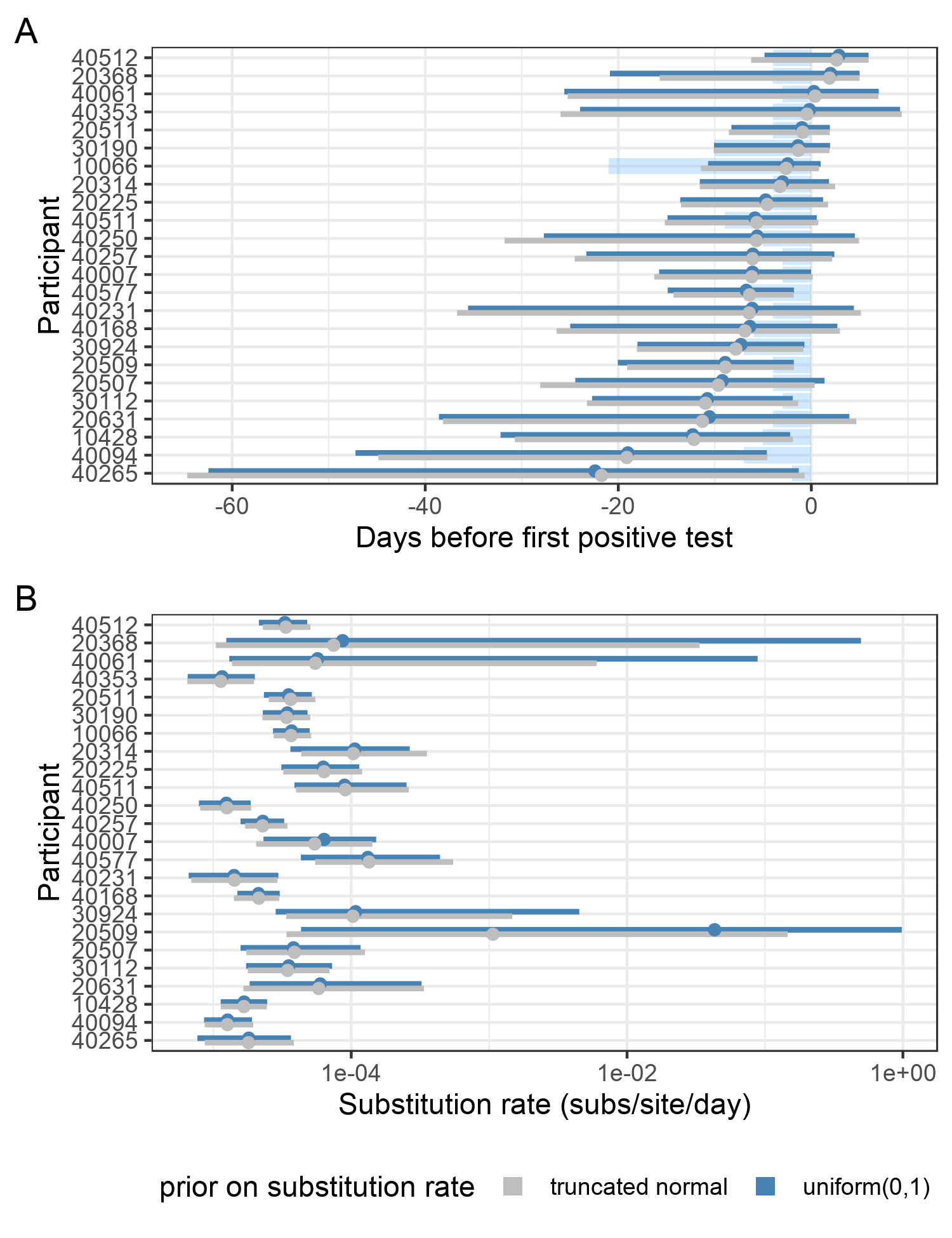

Supplement: S6 Fig — For infections with single founders, BEAST inferences run using either a truncated normal prior (in grey) were compared to a non-informative uniform prior (0,1) (in blue) (both under the best-fitting model). Circles show the median estimates and bars indicate the 95% HPD for the date of infection (A) and the substitution rate (B). The shaded blue area corresponds to the interval between the last negative and first positive HIV-1 RNA test. (TIF) [file ppat.1008179.s007.tif]

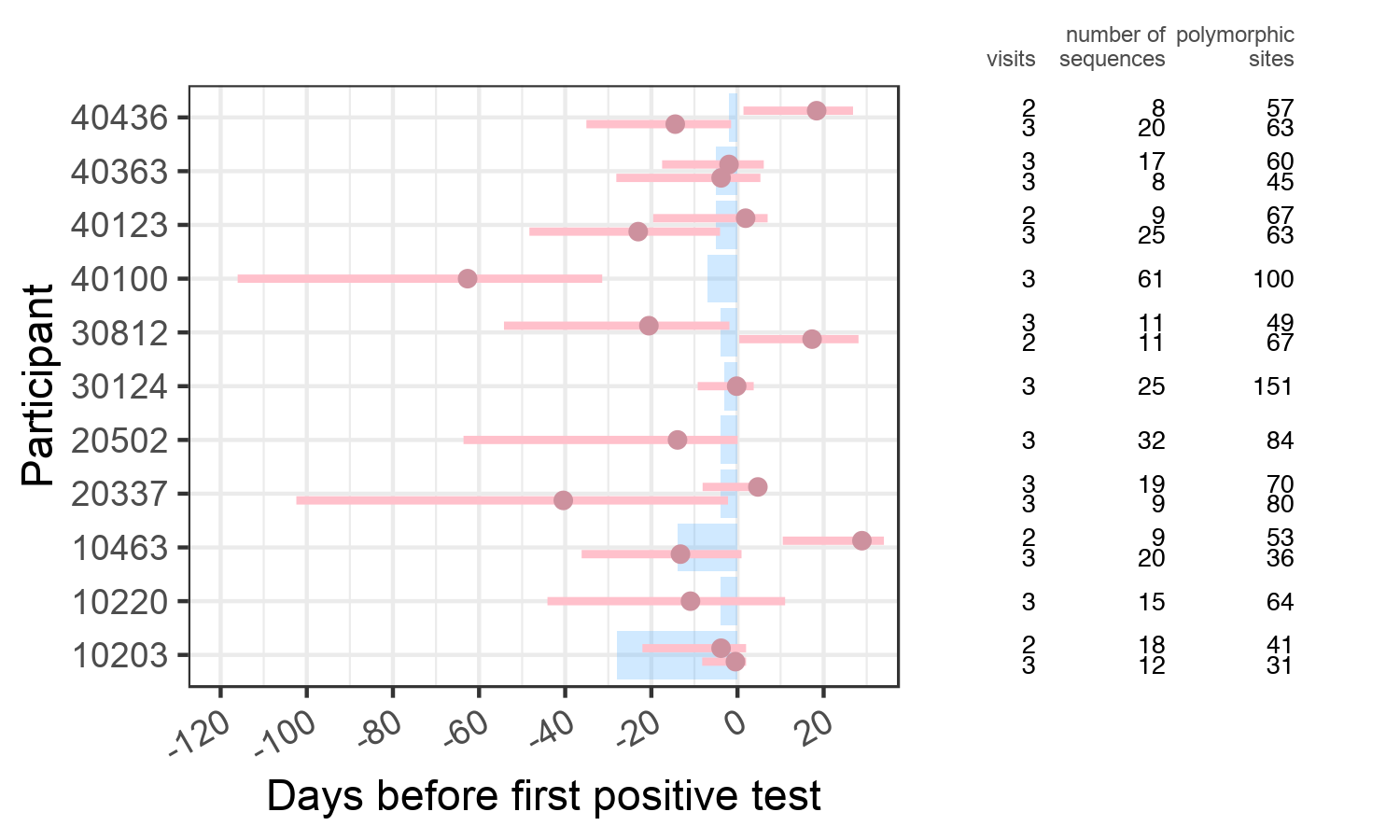

Supplement: S7 Fig — The posterior medians (circle) and 95% highest posterior density interval for the best fitting model are shown. The shaded blue area corresponds to the interval between the last negative and first positive HIV-1 RNA test (or diagnosis date). The number of sequences and number of polymorphic sites corresponding to each subpopulation are reported. Only subpopulations with sequences covering at least two time points, a minimum of five sequences with more than one phylogenetically-informative site, and significant temporal signal were analyzed. (TIF) [file ppat.1008179.s008.tif]

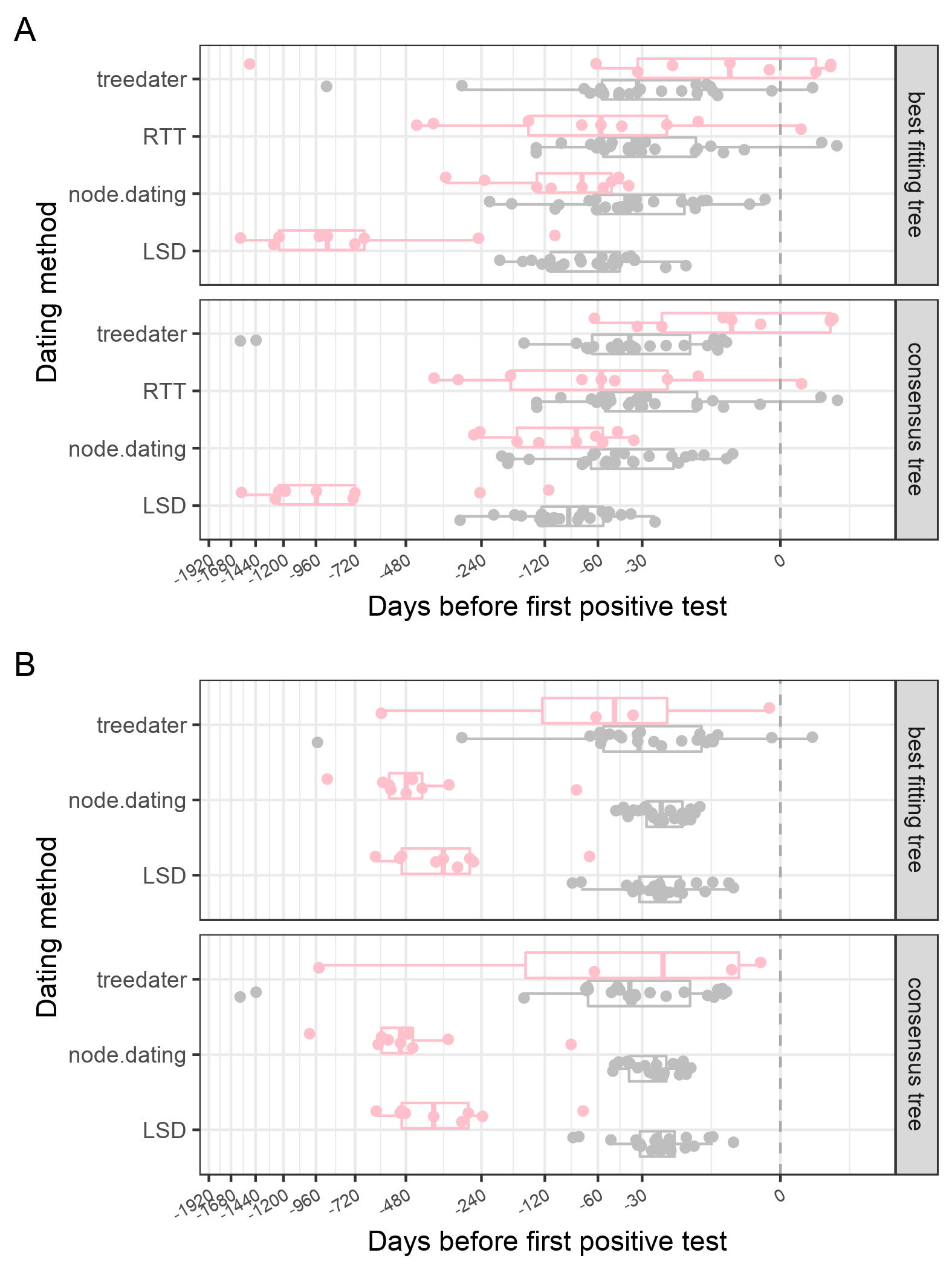

Supplement: S8 Fig — Results based on treedater, RTT, node.dating and LSD are shown for both the best-fitting trees and the consensus from 100 bootstrap trees (using IQ-Tree). In Panel A, no prior information was set; for panel B, we specified the substitution rate as 2.24x10^-5^ for node.dating and LSD and used the truncated normal prior that we used in BEAST for treedater. Infections with single founders are shown in grey and multiple founders in pink. (TIF) [file ppat.1008179.s009.tif]

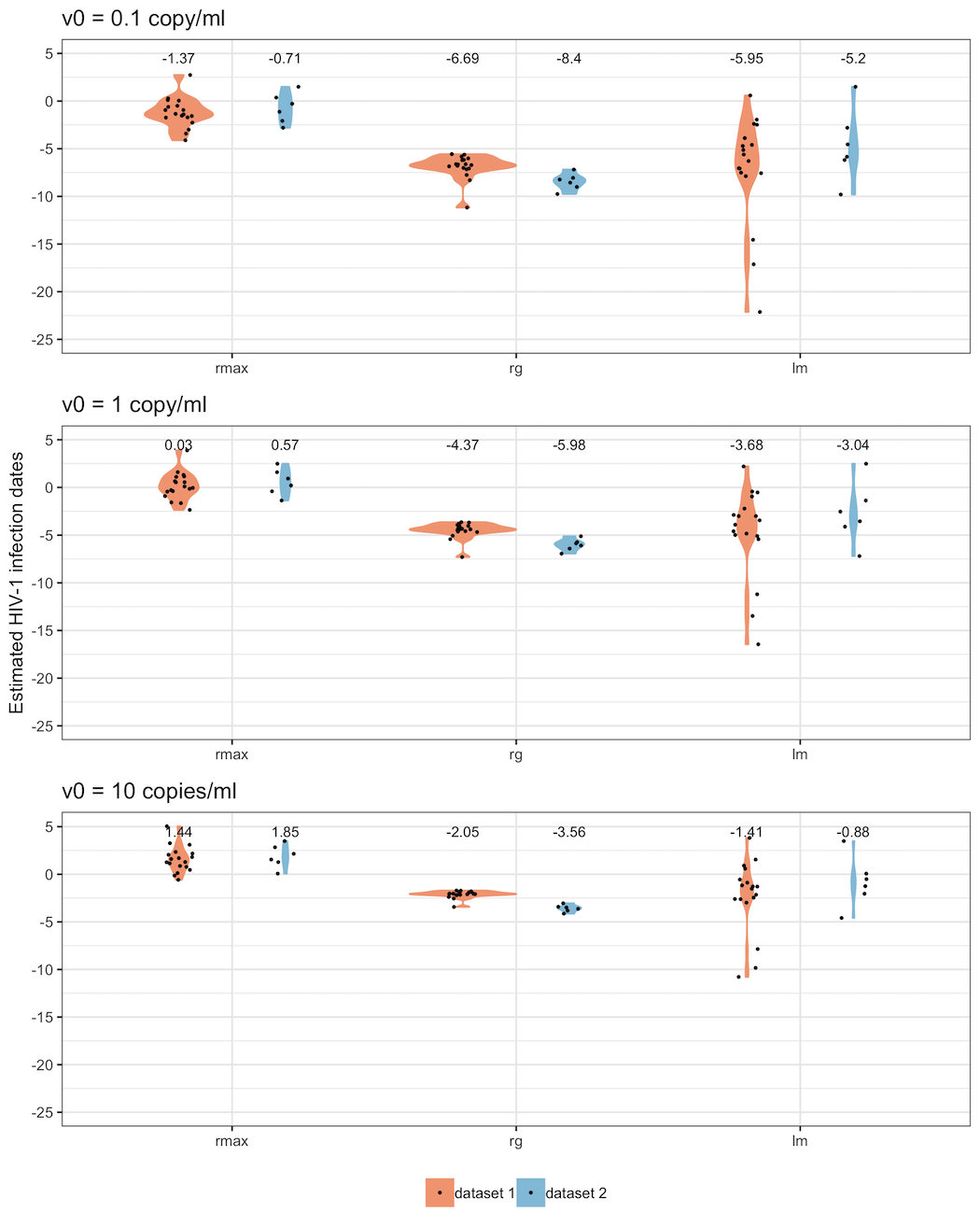

Supplement: S9 Fig — The infection dates were calculated using three methods: highest expansion (rmax), linear mixed-effects model based on data from all subjects (rg), and individual linear models (lm). Viral growth rates were regressed using three initial viral load thresholds: 0.1, 1 and 10 copies/mL. Estimates are compared for two non-human primate (NHP) cohorts with viral load data provided by Drs. Barouch (dataset 1) and Roederer (dataset 2) (animals were infected at day 0). (TIF) [file ppat.1008179.s010.tif]

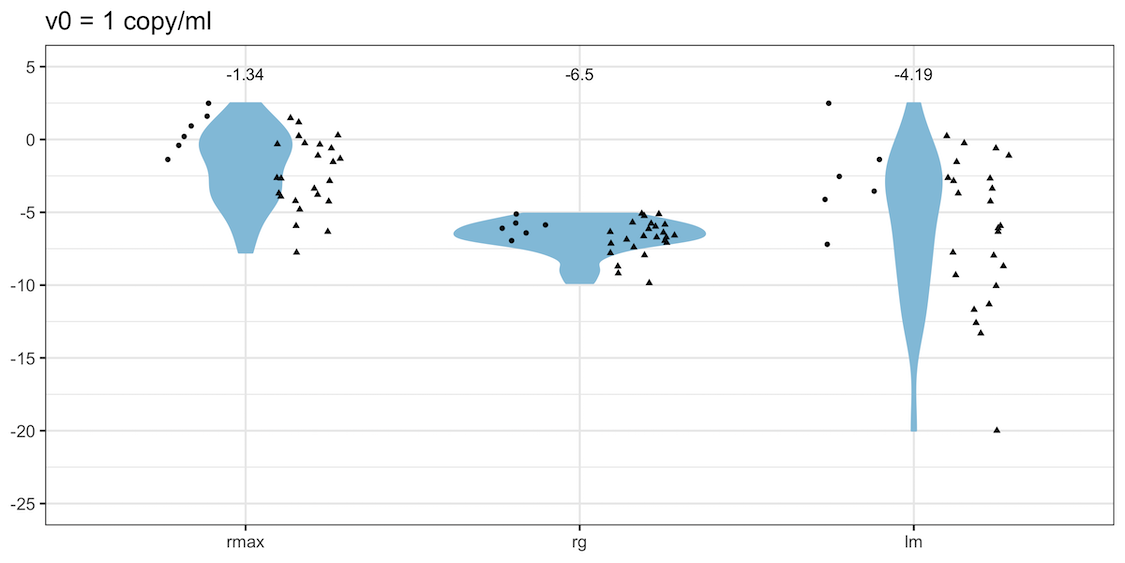

Supplement: S10 Fig — Animals belonged to one of five groups of six animals: four groups received a vaccine (either an Adenovirus or a DNA/Adenovirus regimen administered either intravenously or rectally) or a placebo (Bolton and colleagues [51]). Vaccine or placebo status is figured with a triangle or a circle, respectively. The date when viral loads corresponded to 1 copy per mL of blood was calculated using three methods: highest expansion (rmax), individual linear models (lm) and linear mixed-effects model based on data from all animals (rg). The estimates followed the same rankings whether the animals received a vaccine or a placebo; only the placebo group was reported for the evaluation of methods (S9 Fig). (TIF) [file ppat.1008179.s011.tif]
